# Trace Element Distribution and Arsenic Speciation in Toenails as Affected by External Contamination and Evaluation of a Cleaning Protocol

**DOI:** 10.1021/acs.analchem.3c03962

**Published:** 2024-02-29

**Authors:** Camilla Faidutti, Casey Doolette, Louise Hair, Kim Robin van Daalen, Aliya Naheed, Enzo Lombi, Joerg Feldmann

**Affiliations:** † TESLA, Department of Chemistry, University of Aberdeen, Aberdeen AB24 3UE, U.K.; ‡ Future Industries Institute, University of South Australia, Mawson Lakes, SA 5095, Australia; § TESLA − Analytical Chemistry, Institute of Chemistry, University of Graz, Graz 8010, Austria; ⊥ University of Cambridge, Cambridge CB2 0SP. U.K.; ¶ icddr, b, Dhaka 1212, Bangladesh

## Abstract

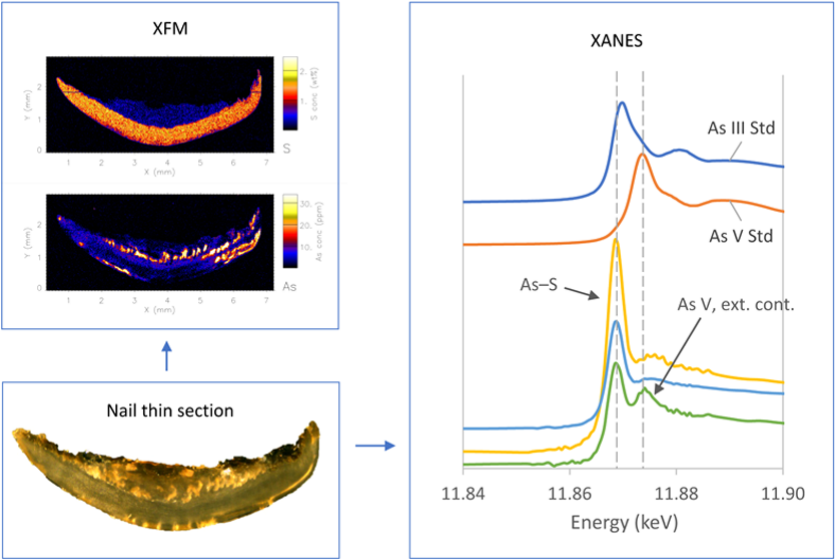

Trace element concentrations in toenail clippings have increasingly been used to measure trace element exposure in epidemeological research. Conventional methods such as inductively coupled plasma mass spectrometry (ICP-MS) and high-performance liquid chromatography ICP-MS (HPLC-ICP-MS) are commonly used to measure trace elements and their speciation in toenails. However, the impact of the removal of external contamination on trace element quantification has not been thoroughly studied. In this work, the microdistribution of trace elements (As, Ca, Co, Cu, Fe, K, Mn, Ni, Rb, S, Sr, Ti, and Zn) in dirty and washed toenails and the speciation of As *in situ* in toenails were investigated using synchrotron X-ray fluorescence microscopy (XFM) and laterally resolved X-ray absorption near edge spectroscopy (XANES). XFM showed different distribution patterns for each trace element, consistent with their binding properties and nail structure. External (terrestrial) contamination was identified and distinguished from the endogenous accumulation of trace elements in toenails—contaminated areas were characterized by the co-occurrence of Co, Fe, and Mn with elements such as Ti and Rb (i.e., indicators of terrestrial contamination). The XANES spectra showed the presence of one As species in washed toenails, corresponding to As bound to sulfhydryl groups. In dirty specimens, a mixed speciation was found in localized areas, containing As^III^–S species and As^V^ species. Arsenic^V^ is thought to be associated with surface contamination and exogenous As. These findings provide new insights into the speciation of arsenic in toenails, the microdistribution of trace elements, and the effectiveness of a cleaning protocol in removing external contamination.

Human biomonitoring has become an increasingly important means to evaluate human exposure to various substances and pollutants and assess potential health risks.^1−3^

The biomonitoring of trace elements is affected by external exposure levels, but it also depends on several other factors, such as chemical characterization, kinetics of absorption, metabolism efficiency, and elimination half-life in the matrix of interest.^4^

In general, the intake of trace elements in the human body can be monitored by measuring their concentrations in different biological matrices, including urine, blood, hair, and nails. Although urine and blood are commonly indicators of short-term trace element exposure, the keratin-rich matrices of hair and nails tend to reflect less recent, long-term exposure.^2^

Numerous environmental and epidemiological studies have used total trace element quantification and speciation techniques in keratin-rich matrices to estimate trace element exposure levels and explore associated health risks.^5−8^ Total elemental quantification (e.g., by inductively coupled plasma mass spectrometry [ICP-MS]) gives an indication of the total internal exposure of trace elements in individuals. However, it does not provide information on the metabolic and biochemical processes associated with each trace element in the human body.^9^ To investigate these aspects, speciation analyses using techniques such as high-performance liquid chromatography ICP-MS (HPLC-ICP-MS) have become particularly useful. Yet, both total trace element quantification and hyphenated MS techniques are sample-destructive (e.g., samples are homogenized through acid digestion) and are not spatially resolved.^10^

In contrast to the aforementioned methods, direct spectroscopic techniques such as synchrotron X-ray fluorescence microscopy (XFM) and X-ray absorption spectroscopy (XAS) minimize sample manipulation and necessitate little or no preparatory steps. In recent years and despite the need for large, dedicated facilities, these techniques have been increasingly applied for monitoring the spatial distributions of several trace elements within samples (XFM) and for spatially resolved speciation analyses (XAS), such as arsenic speciation.^10,11^ Additional imaging and speciation techniques exist, such as laser ablation ICP-MS; however, a detailed comparison of these methods goes beyond the purpose of this study, and more information is widely available in the literature (e.g., a recent review of the analytical methods used for human biomonitoring^12^).

In this study, we applied XFM and X-ray absorption near edge spectroscopy (XANES) on toenails to investigate the suitability of a 4-step cleaning procedure to remove external contamination from toenails, by comparing cleaned versus dirty specimens; to determine the microdistributions of As, Ca, Co, Cu, Fe, K, Mn, Ni, Rb, S, Sr, Ti, and Zn in toenail clippings and their association with the known histology of the nail; and to analyse the speciation of As in toenail clippings, to investigate As incorporation mechanisms and its metabolic transformations.

## Experimental Section

### Sample Collection

Toenail clippings were obtained from 13 participants of the Bangladesh Risk of Acute Vascular Events (BRAVE) study, a hospital-based retrospective case-control study established in 2011 to examine the determinants of acute myocardial infarction in Bangladesh.^13^ Toenail clippings were taken from all ten toes of participants included in the BRAVE study from January 2013 onwards using a clean nail clipper. Clippings were pooled and initially stored in labelled zip-lock bags at room temperature in long-term repositories in both Cambridge (UK) and Dhaka (Bangladesh). All nail samples were then shipped to the University of Aberdeen (UK). Nail clippings from participants selected for this substudy were chosen based on toenail arsenic concentrations, which were obtained from a previous total trace element quantification analysis using ICP-MS. The validity of nails as a biological matrix to investigate As exposure has been previously reported.^14,15^

### Sample Treatment

Two toenail clippings of similar size were taken from each participant (2*n* = 26). For each set, one toenail was not treated, while the other toenail was washed with a 4-step cleaning procedure, using an adapted protocol based on Rodushkin and Axelsson.^16^ Our cleaning protocol included the use of different solvents in sequence to ensure a more effective removal of the chemically different contaminants: acetone (≥99%, Fisher Scientific, UK), Milli-Q water (18 MΩ cm), and 0.5% Triton X-100 (Sigma-Aldrich, UK). Each solution was stirred in an ultrasonic bath for 20 min; at the end of the sequence, the samples were thoroughly rinsed with Milli-Q water, before oven-drying them overnight at 60 °C. The likelihood of nail degradation during the washing procedure was low because nails were washed at room temperature, and no scraping or grinding was applied.

### Sample Preparation

The nail clippings were then embedded in resin (Araldite CY212 Resin, E028 Hard Premix Kit; TAAB, UK). Once hardened, the resin blocks containing the nail clippings were cut into thin sections of 150 μm thickness by using a diamond saw microtome (Leica SP1600, Leica Microsystems Nussloch GmbH, Germany). Multiple thin sections were obtained from each toenail sample (either transversally [perpendicular to the nail bed] or longitudinally [parallel to the nail bed]), and the orientation of the nails was noted before cutting to later identify the histological nail layers in each thin section (i.e., the dorsal [uppermost], the intermediate, and the ventral [closest to the nail bed] nail plate layers). Optical microscope images of each thin section were obtained using a ZEISS Stereo Zoom microscope with a Leica MC170 HD camera. A visual explanation of the sample preparation is given in Figure S1.

### XFM Elemental Mapping and XANES Imaging

Samples were analyzed at the XFM beamline (Australian Synchrotron, Melbourne), where an in-vacuum undulator is used to produce a brilliant X-ray beam. A Si(111) monochromator and Kirkpatrick–Baez mirrors were used to deliver a monochromatic focused beam.^17,18^ The X-ray fluorescence emitted by the specimen was collected using a 384-element Maia detector placed in a backscatter geometry.^19^ For all scans, samples were analyzed continuously in the horizontal direction (“on the fly”). Elemental mapping was conducted at 18.5 keV with a sampling interval of 15 μm in the horizontal direction and a step size of 15 μm in the vertical direction. For all maps, the beam was focused to 2 × 10 μm. The transit time per pixel was 7.5 ms (corresponding to a velocity of 2 mm/s). All spectra were analyzed using GeoPIXE, and images were generated using the Dynamic Analysis method.^20,21^ Elemental concentrations were calculated using the X-ray ionization cross sections of Ebel et al.,^22^ the fundamental parameter database of Elam et al.^23^ extended with PIXE data^24^ and corrections for self-absorption in the sample, absorption in air, and the efficiency response of the detector obtained using standard metal (Mn, Pt) foils; the detected X-ray signals in each pixel are related to calculated model fluoresced X-ray yields for an assumed nail tissue composition and section thickness of 150 μm.

After the elemental maps were collected, fluorescence-XANES imaging was collected on the regions of interest. This consisted of forming “stacks” of fluorescence maps by scanning the entire area of interest 92 times and progressively increasing the energy from 11.802 to 12.117 keV across the As K_α_-edge. This technique allows the extraction of XANES spectra for any area of interest mapped. The energies of the 92 progressive scans were selected on the basis of the features of the XANES spectra of interest. The parameters that were used for fluorescence-XANES imaging were as follows: 10 × 10 μm pixel size and transit time of 10 ms per pixel (i.e., a velocity of 1 mm/s), with the beam focused to 2 × 10 μm.

Two As standards were measured for XANES analysis: NaAsO_2_ (arsenite As^III^) and Na_2_HAsO_4_·7H_2_O (arsenate As^V^). Additionally, data for the As-glutathione standard (As^III^(GS)_3_) were pooled from a library of standards obtained from previous XANES measurements and were used as the reference for As–S bonds. Data for the dimethylarsinic acid (DMA^V^; (CH_3_)_2_AsO_2_H) standard were also obtained from a library of standards. We did not pool other methylated species (dimethylarsinous acid [DMA^III^], monomethylarsonic acid [MMA^V^], and monomethylarsonous acid [MMA^III^]), because, at the energy resolution used by this technique, spectral features cannot easily be used to distinguish between different species.^25^

The GeoPIXE “energy association” module was used to identify the presence of different As species in the samples. The tool shows the relationship between the fluorescence recorded at two different energies near the absorption edge of the species of interest. In the presence of multiple species in the sample, different pixel populations appear on the graph, whereas if only one main species is present, all the pixels tend to align along one direction. RGB images of As–Fe–Ti were also used to identify potential small areas of external contamination otherwise hard to identify using only the “energy association” graphs.

The XANES spectra were extracted from the selected areas and analyzed by Linear Combination Fitting using WinXAS.^26^

## Results and Discussion

### Trace Element Distribution

Transversally, the nail plate consists of three histological layers: the dorsal nail plate (top layer of a few cell layers thick), the intermediate (or middle) nail plate, and the ventral nail plate (the layer closest to the nail bed).^27^ Nail keratins are comprised of 80–90% hard, hair-like α-keratins and 10–20% soft, skin-like epithelial keratins.^28^ Hair-like α-keratin filaments are only present in the intermediate layer of the nail plate, whereas skin-like keratins, poorer in sulfur, are found in the dorsal and ventral layers.^29^ In the intermediate layer, the thiol groups on cysteine (Cys) form stable disulfide bonds (S–S), whereas the dorsal and ventral nail plates have higher levels of free sulfhydryl (thiol) groups.^30^

In this study, XFM was used to map elemental distributions in various toenail thin sections. Compton XFM maps were used to delineate the outlines of the nails in each thin section. Evenly colored Compton maps indicate a uniform thickness of the specimen.

**Figure 1:**
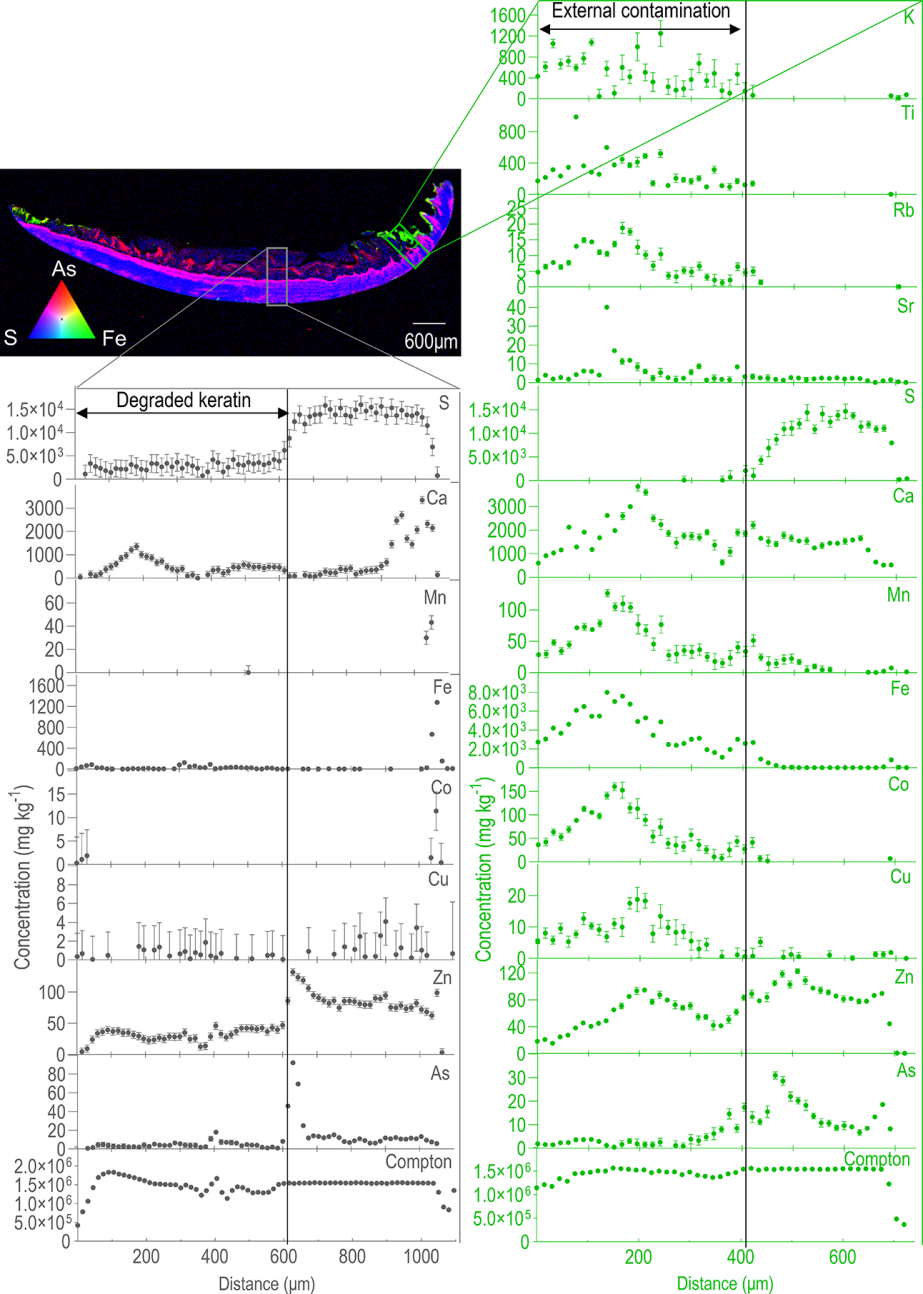
Thin section of the washed nail of participant #8, with corresponding counts profiles across the areas marked in the maps. The green traverse section was drawn in an area with an undulated ventral layer, where the deposition of external contamination is apparent from the higher levels of all trace elements except S, which is absent. K, Ti, and Rb are not found in the nail matrix and instead correlate with Co, Mn, and Fe, in areas corresponding to dirt. The gray traverse section was drawn in an area with degraded keratin.

In washed toenail samples, S was uniformly distributed across the intermediate layer with decreasing concentrations often seen in the dorsal and ventral layers. This can be seen in Figure 1 and Figure S2, which are representative of the samples investigated. Similar to S, Zn was found throughout the washed nail samples with decreasing concentrations from the onset of the dorsal and ventral layers to the external outline. Cu was evenly distributed in all histological layers, generally reflecting the nail Compton outline. Co, Fe, and Mn showed higher concentrations in the dorsal layer. Co, Fe, and Mn were not observed in the intermediate layer. Ca reached its peak concentrations in the dorsal layer, with a more progressive decrease toward the intermediate layer if compared to Co, Mn, and Fe. Ni was mostly very low in concentration and not detectable. Arsenic generally accumulated in the ventral layer. However, in participants with high toenail As concentrations (Table S1), moderate-to-high concentrations could be observed in all three histological layers, with multiple concentration peaks across the nail structure (Figure 1 and Figure S2). In some instances, As was also detected in areas of the dorsal layer with large amounts of Co, Fe, and Mn (Figure S3). In participants with low As concentrations (Table S1), little or no As was observed, as expected.

**Figure 2:**
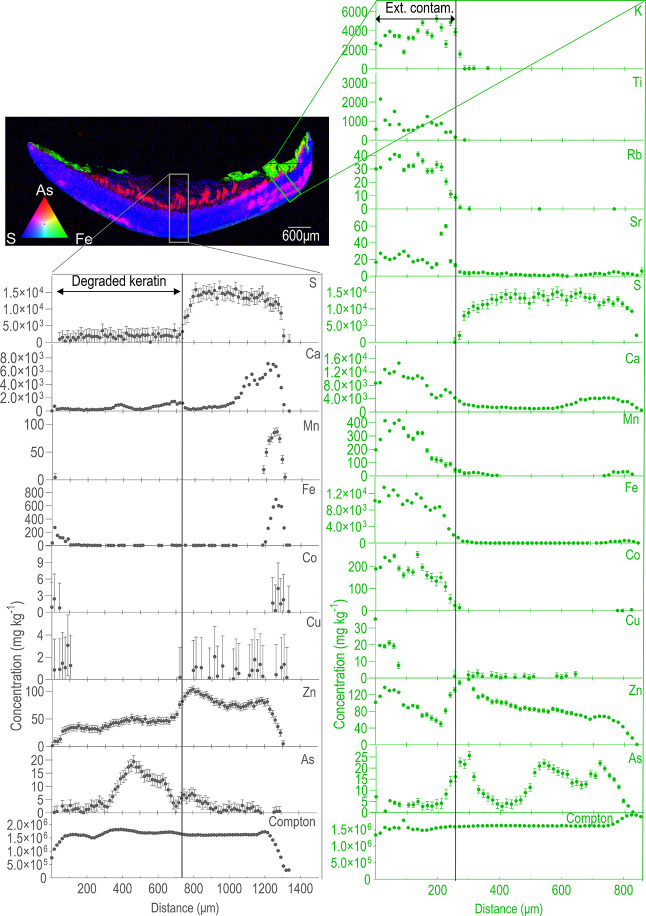
Thin section of the dirty nail of participant #8, with corresponding counts profiles across the areas marked in the maps. The green traverse section was drawn in an area with an undulated ventral layer, where the deposition of external contamination was observed. K, Rb, and Sr are low in concentration, and Ti is not found in the nail matrix. The gray traverse section was drawn in an area with degraded keratin. Counts wise, more contamination is present on the dirty sample, if compared to the washed specimen (Figure 1).

In dirty toenail samples, several trace elements could be observed in concentrated areas in proximity to the ventral layer, while not being a part of the nail structure (Figure 2 and Figure S4). These regions could be differentiated from the keratin outline of the toenail due to the absence of S in the XFM elemental maps. The external contents in the maps were linked to darker regions visible on the whole toenail as well as in the optical microscope images of the thin sections, likely indicating the deposition of exogenous materials onto the nail surface (Figure S5). High amounts of Co, Fe, and Mn were observed in these regions, and in very dirty samples, substantial levels of Ca, Cu, Ni, and Zn were also observed. When detected, As concentrations were not as high as those of other elements in these regions. High concentrations of elements otherwise either absent (Ti) or mostly absent (K, Rb, and Sr) in the nail were also detected in proximity to the nail outline. These latter elements are often found in the environment, and they were used to identify areas of the nail where the deposition of external contamination (e.g., soil and general dirt) may have occurred.

In this study, the observed higher concentrations of S in the intermediate layer are in agreement with the nail composition and previous studies. For instance, Favaro^31^ used microbeam proton induced X-ray emission and observed variation in S along the nail thickness, with the highest concentration in the intermediate layer and lower levels in the dorsal and ventral layers. A significant difference in S content between dorsal–intermediate, and intermediate–ventral, but not between the dorsal and ventral layers was also reported by Mingorance Álvarez et al.,^32^ using scanning electron microscopy with energy-dispersive X-ray spectroscopy.

Given the aforementioned nail structure and composition, we hypothesized that different trace element distribution patterns would exist depending on the chemical binding properties of each trace element. For example, Zn preferentially binds to soft or borderline ligands, such as amino acids with sulfur and nitrogen donor atoms.^33^ In our study, the Zn concentration profile matched that of S, with a decrease in concentration from the onset of the dorsal and ventral layers to the external outline. This finding likely reflects the binding environment in nails previously reported by Katsikini et al.,^34^ where extended X-ray absorption fine structure (EXAFS) results indicated that Zn binds to Cys and His amino-acid residues; with lower S concentrations, Zn concentrations also decrease. Arsenic has a high affinity for sulfhydryl groups and it can bind to cysteine residues in proteins,^35^ as also shown later in this paper. Other trace elements probably have preferred coordination numbers, and different types of amino-acid residues can participate in the coordination.^36^ In our study, higher Ca, Co, Fe, and Mn concentrations were found in the dorsal layer (Figure S6). This area of the nail section is poorer in sulfur, and therefore different amino acids residues other than Cys are likely to be found.

The As distribution in our study was mostly consistent with the literature. XFM mappings of thin sections from previous studies showed discrete layering of arsenic, with propensity for As accumulation in the ventral and dorsal layers rather than in the intermediate layer, and irregular arsenic incorporation along the nail growth axis.^37,38^

### Efficacy of the Cleaning Procedure

Highly concentrated areas of external deposition were consistently washed off following the cleaning procedure when comparing dirty and cleaned toenail samples for each participant (e.g., Figure S7). In some cases, residual exogenous material was still visible after the washing protocol, especially in areas with an undulated ventral layer (e.g., Figure S8). In these instances, however, lower trace element counts were observed compared to the dirty specimens, perhaps indicating that only strongly bound exogenous contamination could not be removed.

The trace elements accumulating endogenously did not seem to be affected by the washing protocol. In fact, the concentration profiles of elements such as As, Ca, Cu, S, and Zn (which all accumulate to different extents within the nail structure) were similar before and after the cleaning steps (Figures 1 and 2 and Figures S2, S4, and S9). Their concentration profiles differed when these elements were building up onto the nail in the form of exogenous particles (Figures 1 and 2 and Figures S2, S4, and S9).

The not-washed samples were visibly very dirty on both the dorsal and ventral nail plates. The ventral layer generally has a more irregular surface, where exogenous contaminants can easily accumulate. Although smoother, the dorsal part is also prone to exposure, for example, in correspondence of ridges and small fissures across the surface. Several sources of contamination are possible, which depend on numerous factors including geographical location, environmental factors, and lifestyle. A detailed analysis of the possible sources of external contamination affecting the toenail samples of the BRAVE study can be found elsewhere.^14^ Briefly, considering the Bangladeshi context of these samples, several sources of external contamination might have affected the trace element contents of the nails, including those of terrestrial, natural origin, and those of anthropogenic origin. Previous evidence also suggests that multiple sources of contamination might exist; Rahman et al.^39^ recently analyzed the surface soil and dust of roadside academic institutions in Dhaka, Bangladesh, and linked Fe, K, Ti, and Sr to mainly natural sources. In a separate study of contamination levels in school dust and soil of Dhaka, several sources were identified, including natural sources for Zr, Fe, Ti, and Rb, traffic-related activities for Cu, Pb, and Zn, and industrial activities (e.g., cement and lime-based mortar dust) for K, Sr, and Ca.^40^ It is thus possible that exogenous materials, such as street dusts and soil, or materials originating from urbanization and industrialization processes, could heavily accumulate on the external layers of the toenails, especially when using open-toed shoes, which is common in Bangladesh.

Overall, the cleaning protocol seemed to be successful at removing external contamination, with dirty specimens having amounts of exogenous contents comparably higher than cleaned toenail clippings (Figure 1 and 2 and Figures S2, S4, S7, and S9). However, several washed samples also showed non-negligible levels of dirt. Residual external contamination is a crucial issue, because sample-destructive analyses do not distinguish between endogenous and exogenous exposure, and therefore high levels of dirt may ultimately affect the suitability of toenails as a proxy to estimate trace elements levels of exposure. In this regard, a separate subanalysis including toenail samples from 211 participants of the BRAVE study showed that the concentrations of several trace elements in washed toenails were affected by external contamination. Large within-subject variabilities were observed for trace elements such as Co (within-subject coefficient of variation [WSCV] 61%, 95% CI 64–69), Fe (58%, 51–65), and Mn (54%, 47–60). Conversely, other elements such as As (19%, 17–20), Cu (25%, 22–28), and Zn (14%, 13–16) had low within-subject variances, suggesting an endogenous build up and low levels of external contamination.^14^

The evidence reported in our study may be helpful to partially address such analytical challenge: the XFM analyses suggest that external contamination of terrestrial origin may be easily distinguished from the endogenous nail contents when analyzing elements such as Ti and Rb. Ti is a nonessential element for humans and does not seem to accumulate within the nail structure. Rb is also a nonessential element and, despite having similar biochemical characteristics to K and potentially substituting for this element in biological samples, the concentration in nails is probably low or negligible. In the future, analyzing Ti and Rb as part of total trace element quantification analyses might provide valuable information on the residual amount of external contamination present onto the nail after the cleaning procedure. Their analysis might also help with the interpretation of other elements in the nails, such as Fe, Co, and Mn, and the nature of their entities (endogenous versus exogenous). K was also found in high concentrations in areas associated with the exogenous build-up, but as it is an essential element, it might not be suitable to establish the presence of external contamination.

### Keratin Degradation

A few analyzed samples showed morphology changes, possibly linked to keratin degradation of the ventral layer. For example, both toenail clippings of participants #8 and #14 were visually thick (Figures S5 and S10). The optical microscope images of the corresponding thin sections showed a rough ventral layer without a uniform structure or tight coherence. Overall, the degraded keratin in the toenail samples we measured was characterized by lower amounts of S and Zn. Ca and Cu were also detected. In participants with high toenail As concentrations (Table S1), high amounts of As were observed in the degraded matrix (if present), representing a major accumulation site (Figure 2).

Keratin degradation can occur as a consequence of several factors, including inflammatory diseases (e.g., psoriasis) and fungal infections (e.g., onychomycosis). Illustratively, onychomycotic nails have higher porosity and lower amounts of disulfide bonds if compared to healthy nails.^41^ In onychomycotic nails, a weakening of the disulfide bond structure occurs due to higher amounts of energetically less stable conformations of the S–S bonds.^42^ Psoriatic nails have also an increased porosity and lower amounts of disulfide bonds.^41,43^ Although the degradation of the disulfide bonds leads to the loss of stability of the nail matrix, contributing to the morphology changes of keratin and making it less compact and less uniform,^44^ it remains unclear whether these changes can affect the results of trace element analyses. Further research is needed to establish whether the presence of inflammatory diseases or fungal infections should be part of the participation exclusion criteria in studies using toenail As to measure (environmental) As exposure.

### Speciation of As

**Figure 3:**
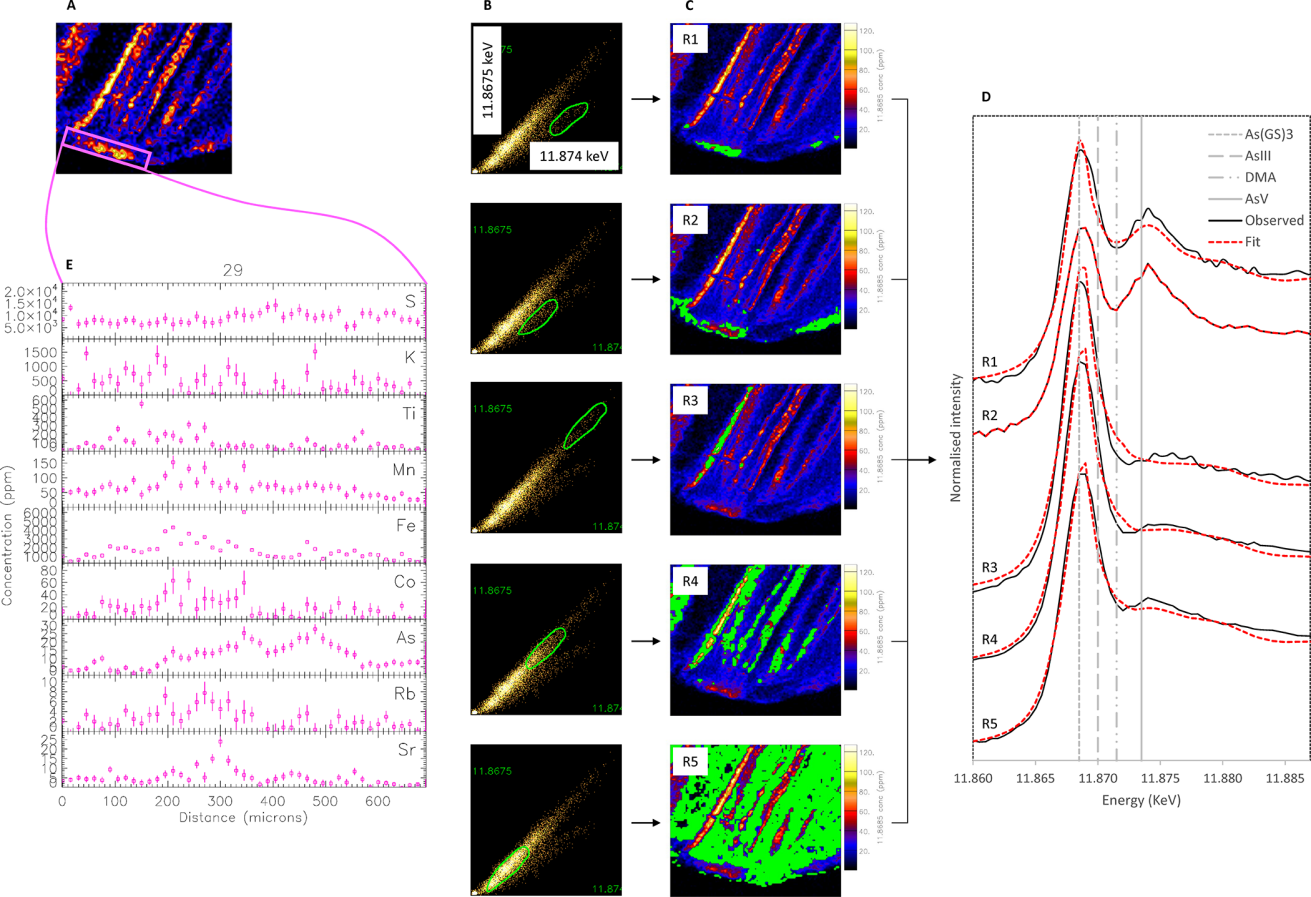
(A) Nail region of participant #29 selected for XANES (dirty sample). (B) Energy association scatter plots corresponding to energies near the white line peaks of the As^III^–S bond in the nail specimen and of the As^V^ standard. (C) Localization of different populations of energy correlations. (D) Extracted XANES for the five different populations; the vertical lines correspond to the white line peaks of As^III^(GS)_3_, As^III^, DMA, and As^V^ standards. (E) Traverse section showing the presence of other trace elements in area R1.

XANES imaging was used to probe the bonding environment of As *in situ*, in localized areas of four thin sections of two participants with moderate-to-high As concentrations in their toenails (Table S1): two thin sections from participant #8 (one dirty, one washed) and two thin sections from participant #29 (one dirty, one washed). The XANES spectra of samples and standards are shown in Figure 3 and Figures S11–S13.

As^III^(GS)_3_ and DMA^V^ were used as reference standards, in addition to As^III^ and As^V^. Glutathione can be used as an analogue of the thiol groups of the nail keratin, binding As through the sulfhydryl group of its Cys residue. DMA^V^ was included as a standard because previous HPLC-ICP-MS speciation analyses showed non-negligible levels of both MMA^V^ and DMA^V^ in the nails of participants #8 and #29 (with the sum of the methylated species being approximately 13% of the total measured As; Figures S14 and S15). Although MMA^V^ was also found in the speciation analyses, it was not included in this study because the white line position of MMA^V^ is very close to the white line of DMA^V^.^25^ At low concentration levels (as is generally the case for the samples measured in this study), the spectra show substantial noise and the two different methylated species are probably not sufficiently resolved. It should be noted that, for the speciation analyses with HPLC-ICP-MS, the nail samples were microwave digested, resulting in the oxidation of any sulfuric species and the conversion of any As^III^ compounds to their As^V^ counterparts. Therefore, we do not know whether the methylated species are bound to S or whether the oxidation state varies. Information on the microwave procedure and the extraction efficiency of methylated species can be found elsewhere.^14,45^ Briefly, the microwave digestion parameters and analysis procedures were optimized to avoid the loss of analytes or As species transformation, ultimately maximizing recoveries while maintaining the stability of the methylated species.^45^

The acquired spectra of the two washed specimens of participants #8 and #29 contained As^III^(GS)_3_ as the dominant As species (accounting for 96–97% of the total As), indicating that As in these samples is likely to be present as As^III^ complexed with thiol residues (Table 1). No spatial variations in As speciation were observed, suggesting the absence of exogenous As (Figure S12).

**Table 1:** Results of Linear Combination Fitting of K-Edge XANES Data Showing Relative Proportions of Arsenic (As) Species for Different Regions of Dirty and Washed Nails for Participants #8 and #29^a^

**sample**	**As**^**III**^**(GS)**_**3**_**(%)**	**As**^**III**^**(%)**	**DMA**^**V**^**(%)**	**As**^**V**^**(%)**	***R* factor**
Washed					
8	97 (2)				0.0045
29	96 (2)				0.0037
Dirty					
8 R1	67 (2)			31 (2)	0.0051
8 R2	79 (2)		10 (3)	8 (3)	0.003
8 R3	98 (2)				0.0033
8 R4	99 (3)				0.0036
8 R5	84 (4)			12 (3)	0.0044
29 R1	62 (2)	12 (2)		25 (3)	0.0046
29 R2	86 (3)		7 (2)		0.0024
29 R3	87 (2)			7 (3)	0.0033
29 R4	94 (2)				0.0041
29 R5	58 (3)			41 (3)	0.0033

a Data are means (SD). *R* factor is a goodness-of-fit estimate.

In the dirty samples, we found mixed speciation (Table 1). The most predominant species was As^III^(GS)_3_, which accounted for 58–99% of the total As, depending on the analyzed sample region. Secondary species included As^III^ (12% in one region only), As^V^ (7–41% in several analyzed regions), and DMA^V^ (7–10% in two separate regions).

The difference in As speciation as related to its distribution is shown in Figure 3 (dirty sample of participant #29) and Figure S13 (dirty sample of participant #8). For participant #29, the data from the energy stack used to generate the As distribution map (Figure 3A) were analyzed using the “energy association” function in GeoPIXE (Figure 3B). Five populations of pixels were selected based on differences in energy correlations, which indicate differences in speciation or As content (areas circled in green in Figure 3B). As^III^ bound to thiol groups is present in all five regions R1–R5, whereas As in dirt is localized at the tip of the nail clipping, in regions R1 and R2. The pink traverse section (Figure 3E) shows the presence of other trace elements (Fe, K, Ti, and Rb) generally associated with external contamination, thus confirming the presence of dirt on the nail. A similar occurrence coinciding with small fractures was observed for the dirty sample of participant #8 (Figure S13), where As was found in Fe-enriched areas (region R4, which corresponds to region R5 identified using RGB images of As–Fe–Ti).

Arsenic was found to readily bind to S. The majority of the acquired spectra indicated the presence of As bound to sulfhydryl groups in the nails. Although As in cleaned samples was present only as As^III^ complexed with thiol residues, we cannot rule out with certainty the presence of other species. With the white line energies of S-containing As species (including methylated species) being similar and not spatially resolved,^25^ we could not establish whether any methylated species were bound to S. Additionally, the fluorescence-XANES imaging was used on limited areas, and there is a possibility that the selected regions did not contain any form of MMA and DMA, perhaps due to a heterogeneous buildup of the methylated As species within the nail samples. In digested samples, the concentrations of MMA and DMA by HPLC-ICP-MS analysis were generally always lower than those of inorganic As, and therefore, there is also a possibility that the concentration levels of the methylated species may have been too low for XANES detection.

In dirty specimens, As^V^ was found in localized areas, highly enriched in Fe, (i.e., likely indicating exogenous As bound to terrestrial contamination). In general, As has an affinity for metal oxides/hydroxides, with Fe-oxides/hydroxides being a major sink for As adsorption—e.g., iron oxides (Fe_2_O_3_), oxide hydroxides (FeOOH), and poorly crystalline ferrihydrite (hydrous ferric oxide).^46,47^ The As^III^ and DMA^V^ species found in a few localized areas of the dirty specimens account for a small proportion of the total As (around 10%). These low estimates cannot however be associated with a high degree of certainty because XANES analyses are inherently insensitive to species present in small proportion, as the XANES spectra are a weighted average of the spectra of all the species present in the area analyzed. Therefore, any estimates around 10% should be interpretated with caution, and definitive conclusions should not be inferred.

Chronic exposure to As has known adverse health effects, causing chronic health impacts.^48^ Previous studies have measured As concentrations in keratin-rich matrices (i.e., hair, fingernails, and toenails) to evaluate the relative exposure of individuals to As. However, As toxicity is also a function of its speciation, and the determination of As chemical species is crucial to understand the fate and toxicity of arsenicals in the human body. Studies using HPLC-ICP-MS employ extraction or digestion techniques that can transform some of the chemical species. Previous studies using this technique identified inorganic and methylated As species in nails but did not report S-bound arsenicals.^49,50^ By contrast, S-bound As species have been detected in nail samples when using XANES spectroscopy. Gault et al.^51^ found that As-glutathione, i.e., S-coordinated As in glutathione, and As^III^–O yielded the best match for the nail samples, with varying proportions between specimens. Pearce et al.^38^ reported a lower oxidation state species, possibly with mixed S and methyl coordination (As^≈III^(−S, −CH_3_)) and a higher oxidation state species (As^≈V^(−O)). Cui et al.^37^ identified S-bonded As species (As–glutathione) as the dominant species, while As^V^ and DMA were observed in smaller amounts across the nail layers; no As^III^ was detected. Ponomarenko et al.^52^ distinguished between: (1) As^III^ type, as a mixture of As bound to thiols, and also to oxygen or methyl groups, with a small contribution from As^V^ species, (2) As^V^ type, fitted by arsenate in aqueous solution, and (3) As^III^ + As^V^ mixture type. Most As^III^ species were best represented by fitting spectra of As-glut and arsenite standards.^52^

These previous findings are variable and do not fully align with the results reported in our study. The results by Cui et al.^37^ were the most similar to our findings, with As-glutathione accounting for 69–76% of the As content in nails. Possible reasons could have contributed to the differences with the other studies. Gault et al.^51^ mentioned problems in the fitting process as a limitation of their analysis, limiting the certainty about the exact species distribution. Pearce et al.^38^ stated that their analyzed samples were sourced from participants who were not exposed to As in seafood or water, but rather As found in soil. By contrast, participants of the BRAVE study were primarily exposed to As through the ingestion of As-rich drinking water and through their diet (e.g., rice). Ponomarenko et al.^52^ found that As-glutathione accounted for only 17–46% of the As content in nails when using As(GS)_3_, As(OH)_3_, and AsO_4_^–^ as standards. The reason for this difference from our results is not clear; however, the authors were also able to produce alternative fittings using standard sets that included inorganic arsenic and methylated species, thus suggesting that other combinations might have been plausible. Further research is therefore required to elucidate the behavior of As species in the nail.

## Conclusions

The microdistribution of trace elements in toenail clippings and the speciation of As in the nail matrix were investigated using XFM mapping and XANES imaging analysis. The findings confirmed the presence of external contamination onto the toenail clippings, which was recognized by identifying areas with co-occurrence of Co, Fe, and Mn with elements such as Ti (i.e., indicators of terrestrial contamination such as soil and dirt). Although the cleaning protocol was shown to successfully remove the majority of dirt, varying levels of residual contamination were still visible in most samples, with residual contamination remaining a persistent experimental and analytical issue. Some elements (e.g., Co, Fe, and Mn) were affected more than others (e.g., As, Cu, and Zn) by this issue, ultimately raising concerns about their suitability for estimating exposure levels. Based on the microdistribution of the measured elements, we suggest including Ti and Rb in future sample-destructive analyses to help estimate the amount of residual external (terrestrial) contamination on the nails after the washing protocol. Although Ti is probably a better proxy of external contamination than Rb, Rb is easier to measure in samples that are acid-digested and analyzed by ICP-MS. However, depending on the origin and nature of the contaminating source, strong associations between Ti and As or Rb and As might not exist, with variability likely preventing the use of Ti or Rb for normalizing out exogenous As contamination (when present). XFM analyses could be used to assess trace element concentrations in specific areas of nail tissues not affected by external contamination.

The result from the XANES analysis showed that As is bound to sulfhydryl groups in the nails when external contamination is absent. In the presence of external contamination, varying levels of As^V^ were also observed, perhaps indicating As^V^ bound to Fe-oxides/hydroxides from geological features. Methylated species were not detected, despite having been quantified in previous HPLC-ICP-MS speciation analyses. Hence, gaps remain in the understanding of the As incorporation mechanisms and its metabolic transformations, and further studies are required to better elucidate these aspects.

In several samples, morphology changes were observed in the keratin structure, possibly as a result of a disease. Future studies should investigate whether morphology changes could impact the overall trace element analyses.

## Supporting Information Available

This file contains further information on the analyzed toenails, including measured ICP-MS concentrations, a visual explanation of the sample preparation, additional XFM and XANES images, and HPLC-ICP-MS results (PDF)

## References

[ref1] Angerer J.; Ewers U.; Wilhelm M. Human biomonitoring: state of the art. Int. J. Hyg. Environ. Health 2007, 210, 201–228. 10.1016/j.ijheh.2007.01.024. 17376741

[ref2] Gil F.; Hernández A. F. Toxicological importance of human biomonitoring of metallic and metalloid elements in different biological samples. Food Chem. Toxicol. 2015, 80, 287–297. 10.1016/j.fct.2015.03.025. 25837421

[ref3] Waseem A.; Arshad J. A review of human biomonitoring studies of trace elements in Pakistan. Chemosphere 2016, 163, 153–176. 10.1016/j.chemosphere.2016.08.011. 27529382

[ref4] Aylward L. L.; Hays S. M.; Smolders R.; et al. Sources of variability in biomarker concentrations. J. Toxicol. Environ. Health, Part B 2014, 17, 45–61. 10.1080/10937404.2013.864250. 24597909

[ref5] Gutiérrez-González E.; García-Esquinas E.; de Larrea-Baz N. F.; Salcedo-Bellido I.; Navas-Acien A.; Lope V.; Gómez-Ariza J. L.; Pastor R.; Pollán M.; Pérez-Gómez B.; et al. Toenails as biomarker of exposure to essential trace elements: a review. Environ. Res. 2019, 179, 108787 10.1016/j.envres.2019.108787. 31610392 PMC8164381

[ref6] Kempson I. M.; Lombi E. Hair analysis as a biomarker for toxicology, disease and health status. Chem. Soc. Rev. 2011, 40, 3915–3940. 10.1039/c1cs15021a. 21468435

[ref7] Salcedo-Bellido I.; Gutiérrez-González E.; García-Esquinas E.; et al. Toxic metals in toenails as biomarkers of exposure: a review. Environ. Res. 2021, 197, 111028 10.1016/j.envres.2021.111028. 33753073

[ref8] Signes-Pastor A. J.; Gutiérrez-González E.; García-Villarino M.; et al. Toenails as biomarker of exposure to arsenic: a review. Environ. Res. 2021, 195, 110286 10.1016/j.envres.2020.110286. 33075355 PMC7987585

[ref9] Caruso J. A.; Klaue B.; Michalke B.; Rocke D. M. Group assessment: elemental speciation. Ecotoxicol. Environ. Saf. 2003, 56, 32–44. 10.1016/S0147-6513(03)00048-4. 12915138

[ref10] Feldmann J.; Salaün P.; Lombi E. Critical review perspective: elemental speciation analysis methods in environmental chemistry—moving towards methodological integration. Environ. Chem. 2009, 6, 275–289. 10.1071/EN09018.

[ref11] Gräfe M.; Donner E.; Collins R. N.; Lombi E. Speciation of metal(loid)s in environmental samples by X-ray absorption spectroscopy: a critical review. Anal. Chim. Acta 2014, 822, 1–22. 10.1016/j.aca.2014.02.044. 24725743

[ref12] Lum J.T.-S.; Chan Y.-N.; Leung K S.-Y. Current applications and future perspectives on elemental analysis of non-invasive samples for human biomonitoring. Talanta 2021, 234, 122683 10.1016/j.talanta.2021.122683. 34364482

[ref13] Chowdhury R.; Alam D. S.; Fakir I. I.; et al. The Bangladesh Risk of Acute Vascular Events (BRAVE) study: objectives and design. Eur. J. Epidemiol. 2015, 30, 577–587. 10.1007/s10654-015-0037-2. 25930055 PMC4516898

[ref14] Faidutti C. Trace elements in toenails in environmental epidemiology. PhD thesis, University of Aberdeen, 2022.

[ref15] Slotnick M. J.; Nriagu J. O. Validity of human nails as a biomarker of arsenic and selenium exposure: a review. Environ. Res. 2006, 102, 125–139. 10.1016/j.envres.2005.12.001. 16442520

[ref16] Rodushkin I.; Axelsson M. D. Application of double focusing sector field ICP-MS for multielemental characterization of human hair and nails. Part I. Analytical methodology. Sci. Total Environ. 2000, 250, 83–100. 10.1016/S0048-9697(00)00369-7. 10811254

[ref17] Paterson D. J.; Boldeman J. W.; Cohen D. D.; Ryan C. G. Microspectroscopy beamline at the Australian synchrotron. AIP Conf. Proc. 2007, 879, 864–867. 10.1063/1.2436197.

[ref18] Paterson D. J.; de Jonge M. D.; Howard D. L.; et al. The X-ray fluorescence microscopy beamline at the Australian synchrotron. AIP Conf. Proc. 2011, 1365, 219–222. 10.1063/1.3625343.

[ref19] Kirkham R.; Dunn P. A.; Kuczewski A. J.; et al. The Maia spectroscopy detector system: Engineering for integrated pulse capture, low-latency scanning and real-time processing. AIP Conf. Proc. 2010, 1234, 240–243. 10.1063/1.3463181.

[ref20] Ryan C. G. Quantitative trace element imaging using PIXE and the nuclear microprobe. Int. J. Imaging Syst. Technol. 2000, 11, 219–230. 10.1002/ima.1007.

[ref21] Ryan C. G.; Jamieson D. N. Dynamic analysis: on-line quantitative PIXE microanalysis and its use in overlap-resolved elemental mapping. Nucl. Instrum. Methods Phys. Res., Sect. B 1993, 77, 203–214. 10.1016/0168-583X(93)95545-G.

[ref22] Ebel H.; Svagera R.; Ebel M. F.; Shaltout A.; Hubbell J. H. Numerical description of photoelectric absorption coefficients for fundamental parameter programs. X-Ray Spectrom. 2003, 32, 442–451. 10.1002/xrs.667.

[ref23] Elam W. T.; Ravel B. D.; Sieber J. R. A new atomic database for X-ray spectroscopic calculations. Radiat. Phys. Chem. 2002, 63, 121–128. 10.1016/S0969-806X(01)00227-4.

[ref24] Ryan C. G.; Etschmann B. E.; Vogt S.; et al. Nuclear microprobe-synchrotron synergy: towards integrated quantitative real-time elemental imaging using PIXE and SXRF. Nucl. Instrum. Methods Phys. Res. 2005, B231, 183–188. 10.1016/j.nimb.2005.01.054.

[ref25] Smith P. G.; Koch I.; Gordon R. A.; Mandoli D. F.; Chapman B. D.; Reimer K. J. X-ray absorption near-edge structure analysis of arsenic species for application to biological environmental samples. Environ. Sci. Technol. 2005, 39, 248–254. 10.1021/es049358b. 15667101

[ref26] Ressler T. WinXAS: a pogram for X-ray absorption spectroscopy data analysis under MS-Windows. J. Synchrotron Radiat. 1998, 5, 118–122. 10.1107/S0909049597019298. 16687813

[ref27] Wang B.; Yang W.; McKittrick J.; Meyers M. A. Keratin: Structure, mechanical properties, occurrence in biological organisms, and efforts at bioinspiration. Prog. Mater. Sci. 2016, 76, 229–318. 10.1016/j.pmatsci.2015.06.001.

[ref28] Lynch M. H.; O’Guin W. M.; Hardy C.; Mak L.; Sun T. T. Acidic and basic hair/nail (“hard”) keratins: their colocalization in upper cortical and cuticle cells of the human hair follicle and their relationship to “soft” keratins. J. Cell Biol. 1986, 103, 2593–2606. 10.1083/jcb.103.6.2593. 2432071 PMC2114622

[ref29] Garson J. C.; Baltenneck F.; Leroy F.; Riekel C.; Müller M. Histological structure of human nail as studied by synchrotron X-ray microdiffraction. Cell Mol. Biol. (Noisy-le-grand) 2000, 46, 1025–1034. 10976860

[ref30] De Berker D. A. R., Ruben B. S., Baran R. Science of the nail apparatus. In: Baran R., de Berker D. A. R., Holzberg M., Piraccini B. M., Richert B., Thomas L. eds. Baran & Dawber’s Diseases of the Nails and their Management. 5th ed. John Wiley and Sons Ltd: Hoboken, US, 2019, 1–58.

[ref31] Favaro P. C. Metrology of nail clippings as trace element biomarkers. PhD thesis, Delft University of Technology, 2013.

[ref32] Mingorance Álvarez E.; Martínez Quintana R.; Pérez Pico A. M.; Mayordomo R. Predictive model of nail consistency using scanning electron microscopy with energy-dispersive X-ray. Biology (Basel). 2021, 10, 53 10.3390/biology10010053. 33445794 PMC7828269

[ref33] Brylinski M.; Skolnick J. FINDSITE-metal: Integrating evolutionary information and machine learning for structure-based metal binding site prediction at the proteome level. Proteins 2011, 79, 735–751. 10.1002/prot.22913. 21287609 PMC3060289

[ref34] Katsikini M.; Mavromati E.; Pinakidou F.; Paloura E. C.; Gioulekas D. Zn-K edge EXAFS study of human nails. J. Phys.: Conf. Ser. 2009, 190, 012204 10.1088/1742-6596/190/1/012204.

[ref35] Shen S.; Li X. F.; Cullen W. R.; Weinfeld M.; Le X. C. Arsenic binding to proteins. Chem. Rev. 2013, 113, 7769–7792. 10.1021/cr300015c. 23808632 PMC3797521

[ref36] Dokmanić I.; Šikić M.; Tomić S. Metals in proteins: correlation between the metal-ion type, coordination number and the amino-acid residues involved in the coordination. Acta Crystallogr., Sect. D: Biol. Crystallogr. 2008, 64, 257–263. 10.1107/S090744490706595X. 18323620

[ref37] Cui J.; Shi J.; Jiang G.; Jing C. Arsenic levels and speciation from ingestion exposures to biomarkers in Shanxi, China: implications for human health. Environ. Sci. Technol. 2013, 47, 5419–5424. 10.1021/es400129s. 23600923

[ref38] Pearce D. C.; Dowling K.; Gerson A. R.; et al. Arsenic microdistribution and speciation in toenail clippings of children living in a historic gold mining area. Sci. Total Environ. 2010, 408, 2590–2599. 10.1016/j.scitotenv.2009.12.039. 20067849

[ref39] Rahman M. S.; Kumar P.; Ullah M.; et al. Elemental analysis in surface soil and dust of roadside academic institutions in Dhaka city, Bangladesh and their impact on human health. Environ. Chem. Ecotoxicol. 2021, 3, 197–208. 10.1016/j.enceco.2021.06.001.

[ref40] Rahman M. S.; Kumar S.; Nasiruddin M.; Saha N. 2021b. Deciphering the origin of Cu, Pb, and Zn contamination in school dust and soil of Dhaka, a megacity in Bangladesh. Environ. Sci. Pollut. Res. 2021, 28 (28), 40808–40823. 10.1007/s11356-021-13565-7. 33772469

[ref41] Cutrín Gómez E.; Anguiano Igea S.; Delgado-Charro M. B.; Gómez Amoza J. L.; Otero Espinar F. J. Microstructural alterations in the onychomycotic and psoriatic nail: relevance in drug delivery. Eur. J. Pharm. Biopharm. 2018, 128, 48–56. 10.1016/j.ejpb.2018.04.012. 29673870

[ref42] Wen W.; Meng Y.; Xiao J.; Zhang P.; Zhang H. Comparative study on keratin structural changes in onychomycosis and normal human finger nail specimens by Raman spectroscopy. J. Mol. Struct. 2013, 1038, 35–39. 10.1016/j.molstruc.2013.01.051.

[ref43] Chiriac A. E.; Azoicai D.; Coroaba A.; et al. Raman spectroscopy, X-ray diffraction, and scanning electron microscopy as noninvasive methods for microstructural alterations in psoriatic nails. Molecules 2021, 26, 280 10.3390/molecules26020280. 33429943 PMC7826832

[ref44] Coroaba A.; Pinteala T.; Chiriac A.; Chiriac A. E.; Simionescu B. C.; Pinteala M. Degradation mechanism induced by psoriasis in human fingernails: a different approach. J. Invest. Dermatol. 2016, 136, 311–313. 10.1038/JID.2015.387. 26763451

[ref45] Hair L. Arsenic speciation in toenails as a biomarker of arsenic exposure and indicator of cardiovascular disease. PhD thesis, University of Aberdeen, 2021.

[ref46] Jang Y. C.; Somanna Y.; Kim H. Source, distribution, toxicity and remediation of arsenic in the environment—a review. Int. J. Applied Environ. Sci. 2016, 11, 559–581.

[ref47] Sherman D. M.; Randall S. R. Surface complexation of arsenic(V) to iron(III) (hydr)oxides: Structural mechanism from ab initio molecular geometries and EXAFS spectroscopy. Geochim. Cosmochim. Acta 2003, 67, 4223–4230. 10.1016/S0016-7037(03)00237-0.

[ref48] Ahmad S. A., Khan M. H. 2—Ground water arsenic contamination and its health effects in Bangladesh. In: Flora S. J. S. ed. Handbook of Arsenic Toxicology. Academic Press, 2015, 51–72.

[ref49] Button M.; Jenkin G. R. T.; Harrington C. F.; Watts M. J. Human toenails as a biomarker of exposure to elevated environmental arsenic. J. Environ. Monit. 2009, 11, 610–617. 10.1039/b817097e. 19280039

[ref50] Mandal B. K.; Ogra Y.; Anzai K.; Suzuki K. T. Speciation of arsenic in biological samples. Toxicol. Appl. Pharmacol. 2004, 198, 307–318. 10.1016/j.taap.2003.10.030. 15276410

[ref51] Gault A. G.; Rowland H. A. L.; Charnock J. M.; et al. Arsenic in hair and nails of individuals exposed to arsenic-rich groundwaters in Kandal province. Cambodia. Sci. Total Environ. 2008, 393, 168–176. 10.1016/j.scitotenv.2007.12.028. 18234288

[ref52] Ponomarenko O.; Gherase M. R.; LeBlanc M. S.; et al. Synchrotron X-ray absorption spectroscopy analysis of arsenic chemical speciation in human nail clippings. Environ. Chem. 2014, 11, 632–643. 10.1071/EN13240.

