# Supplementary material for: Trace Element Distribution and Arsenic Speciation in Toenails as Affected by External Contamination and Evaluation of a Cleaning Protocol
Source: Anal Chem. 2024 Feb 29;96(10):4039–47. doi: 10.1021/acs.analchem.3c03962 (PMC10938283; doi:10.1021/acs.analchem.3c03962)
Supplement: Supplementary file 1 — ac3c03962_si_001.pdf [file ac3c03962_si_001.pdf]

Supporting Information

Trace Element Distribution and Arsenic Speciation in Toenails as Affected by External Contamination and Evaluation of a Cleaning Protocol

Camilla Faidutti[a], Casey Doolette[b], Louise Hair[a], Kim Robin van Daalen[c,d], Aliya Naheed[e], Enzo Lombi[b], and Joerg Feldmann[f]*

Affiliations:

[a] TESLA, Department of Chemistry, University of Aberdeen, Aberdeen AB24 3UE, UK

[b] Future Industries Institute, University of South Australia, Mawson Lakes, SA 5095, Australia

[c] British Heart Foundation Cardiovascular Epidemiology Unit, Department of Public Health and Primary Care, University of Cambridge, Cambridge CB2 0BB, UK

[d] Victor Phillip Dahdaleh Heart and Lung Research Institute, University of Cambridge, Cambridge CB2 0BB, UK

[e] Non Communicable Diseases, Nutrition Research Division, icddr,b, Dhaka 1212, Bangladesh

[f] TESLA – Analytical Chemistry, Institute of Chemistry, University of Graz, 8010 Graz, Austria

*Email: joerg.feldmann@uni-graz.at

Table of contents

Table S1. As concentrations (mg/kg) in washed toenail samples of the 13 selected participants. (p 2)

Figure S1. Visual explanation of the sample preparation. (p 3)

Figure S2. XFM maps of the measured trace elements in a thin section of the washed nail of participant #8. (p 4)

Figure S3. Example of As binding types. (p 5)

Figure S4. XFM maps of the measured trace elements in a thin section of the dirty nail of participant #8. (p 6)

Figure S5. a) Whole toenails from participant #8. b) Optical microscope image of two thin sections. c) RGB images for samples 8b3 and 8a2, showing Co (red), S (green), and Fe (blue). (p 7)

Figure S6. Traverse section with a visible distinction between endogenous and exogenous incorporation of Co, Mn, and Fe. (p 8)

Figure S7. XFM maps of the measured trace elements in a thin section of the nails of participant #24 (dirty, left versus washed, right). (p 9)

Figure S8. XFM maps of the measured trace elements in a thin section of the washed nail of participant #15. (p 10)

Figure S9. XFM maps for multiple transversal thin sections of participant #8. (pp 11–13)

Figure S10. Longitudinal thin sections. (p 14)

Figure S11. As K-edge XANES spectra of the four standards used for analysis. (p 15)

Figure S12. XANES analysis—participants #29 and #8 (washed samples). (p 16)

Figure S13. XANES analysis—participant #8 (dirty sample). (p 17)

Figure S14. HPLC-ICPMS chromatogram showing the As content in participant #8. (p 18)

Figure S15. HPLC-ICPMS chromatogram showing the As content in participant #29. (p 18)

References (p 18)

Table S1. As concentrations (mg/kg) in washed toenail samples of the 13 selected participants.

**Participant ID**	**As, ppm**
3	0.26
12	0.46
13	0.50
24	0.79
28	0.99
18	1.82
9	2.08
5	3.26
14	5.31
15	5.32
29	6.71
30	8.46
8	12.84

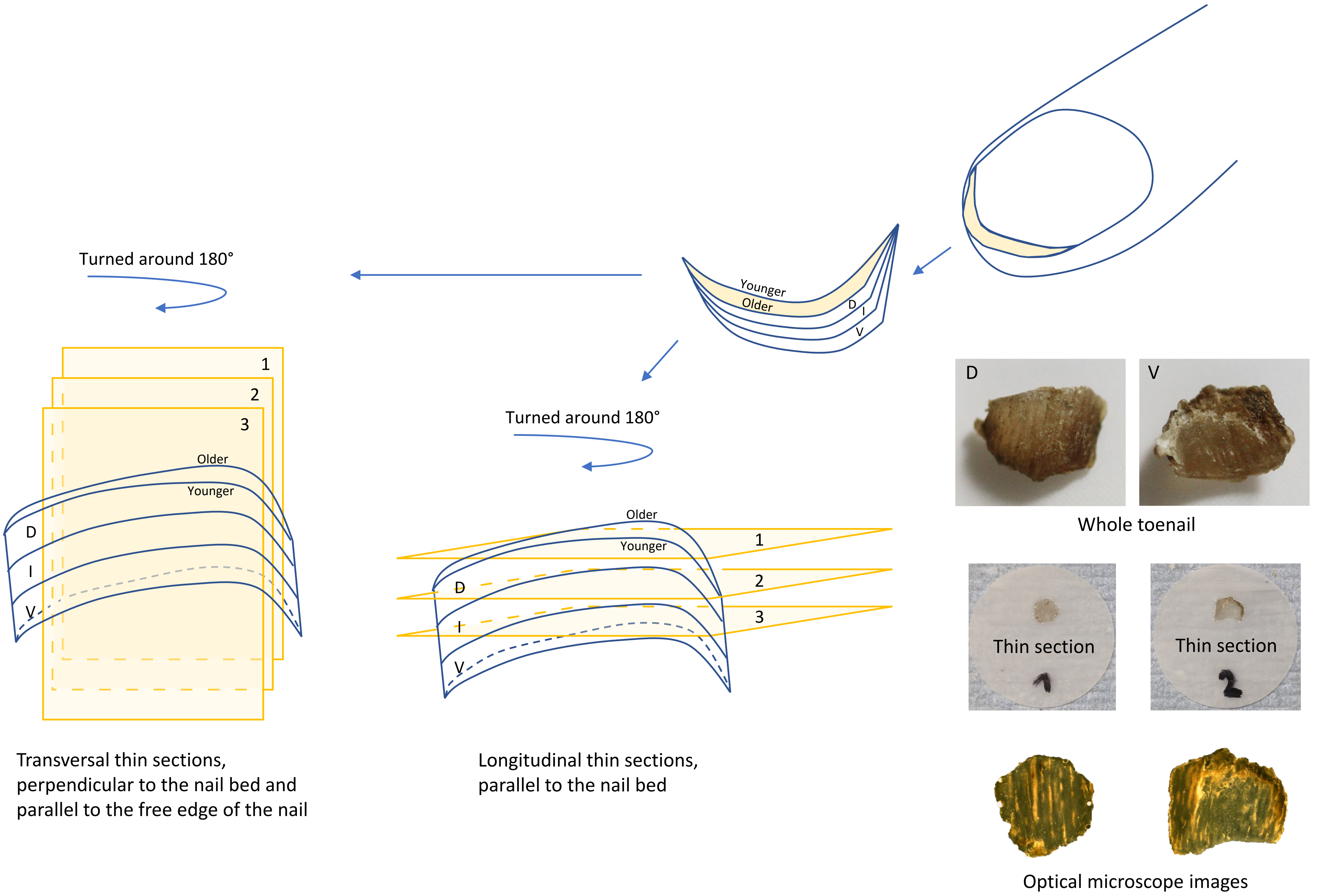

Figure S1. Visual explanation of the sample preparation. D=dorsal. I=intermediate. V=ventral.

#8 (washed)

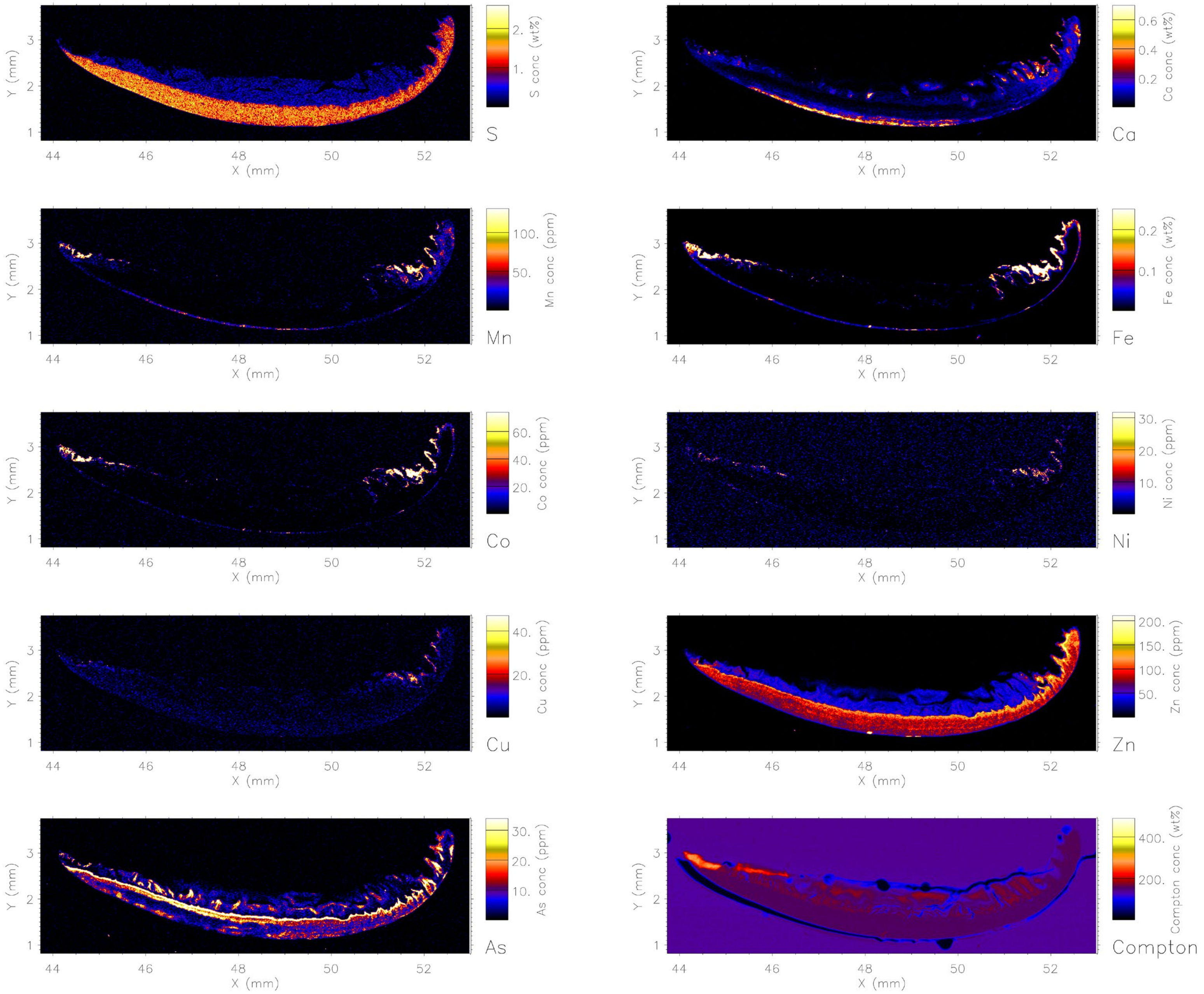

Figure S2. XFM maps of the measured trace elements in a thin section of the washed nail of participant #8, showing the dorsal (external arc), intermediate, and ventral (inner arc) layers. S is uniformly distributed across the nail, with a drop in concentration at the onset of the degraded keratin.

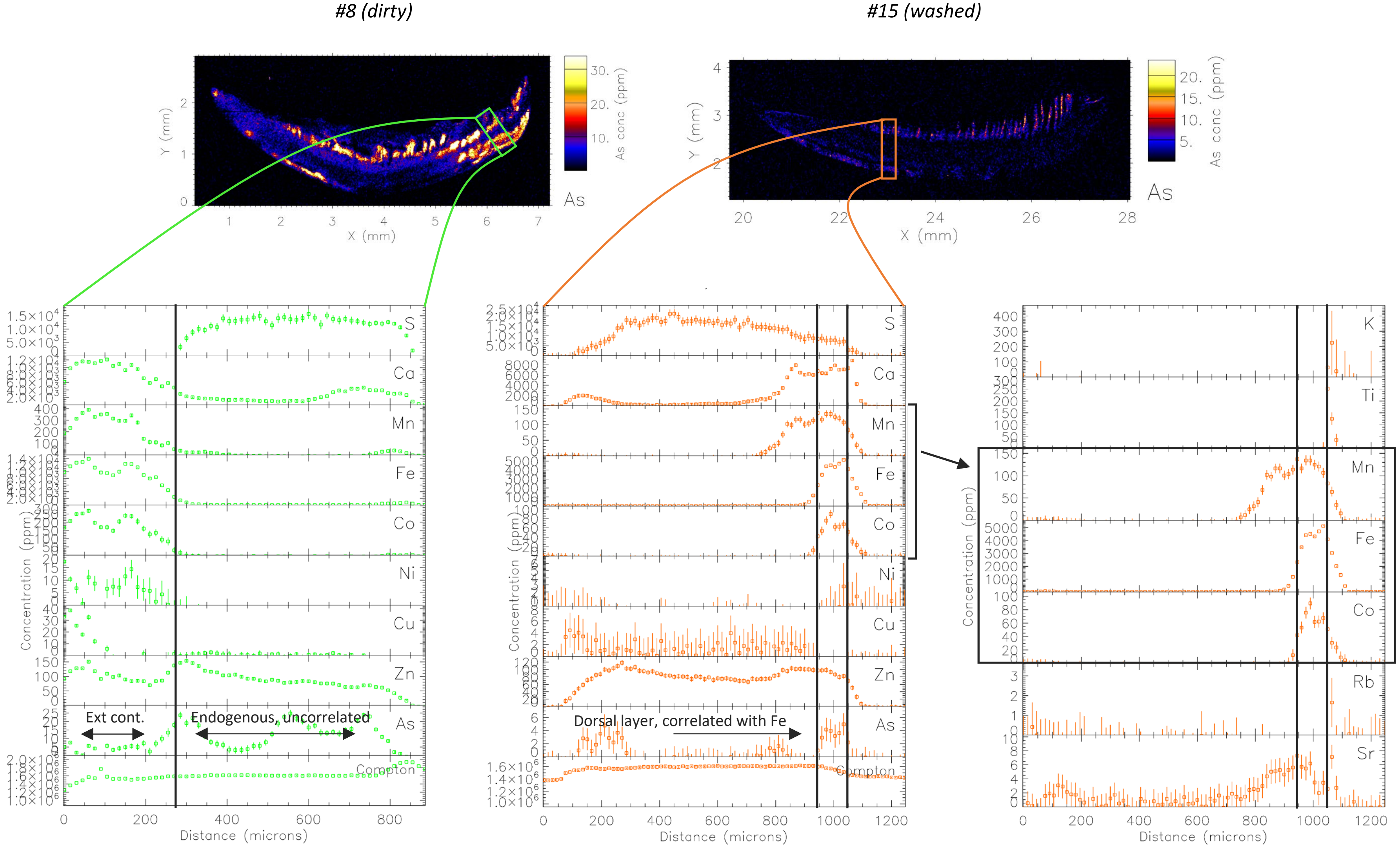

Figure S3. Example of As binding types: present in the external contamination, accumulating independently from the other trace elements, and As peak coinciding with Fe.

#8 (dirty)

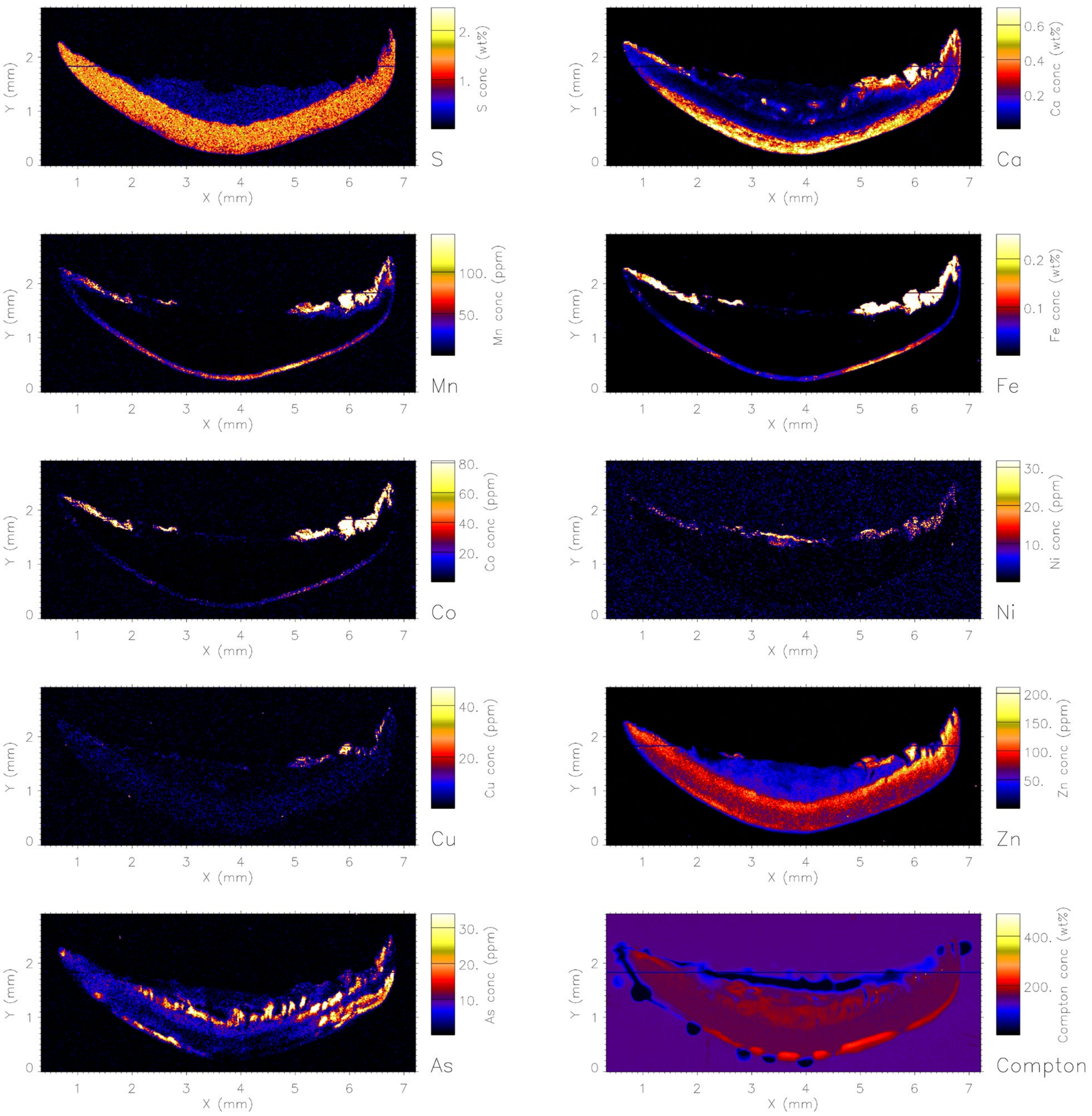

Figure S4. XFM maps of the measured trace elements in a thin section of the dirty nail of participant #8, showing the dorsal (external arc), intermediate, and ventral (inner arc) layers.

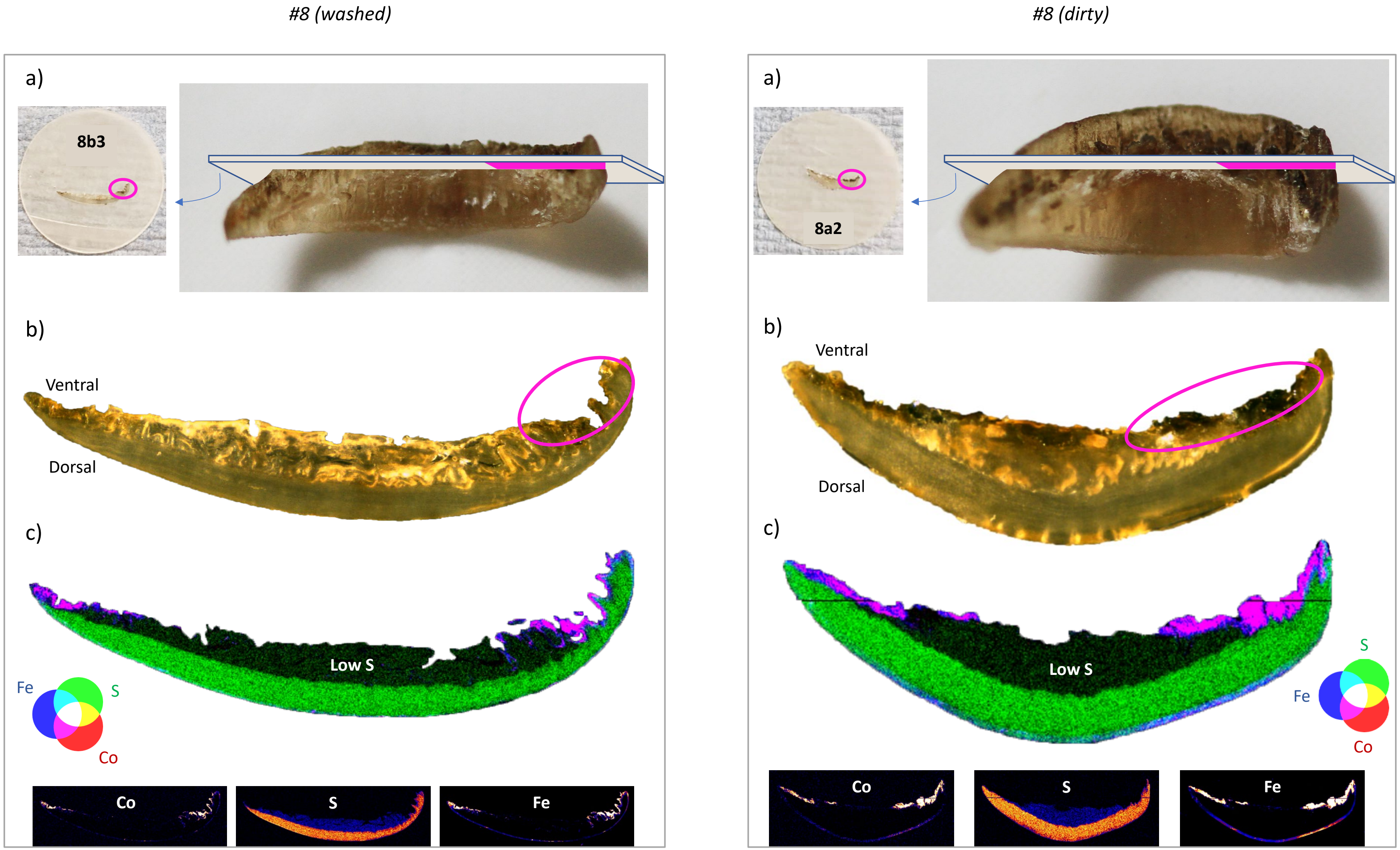

Figure S5. a) Whole toenails from participant #8 (washed, left panel versus dirty, right panel), with their ventral layers facing up. The thin sections were obtained by cutting the nail transversally, to obtain 150µm-thick sections that included all histological layers: dorsal (external arc), intermediate, and ventral (inner arc).

b) Optical microscope image of two thin sections (washed, left panel versus dirty, right panel). A morphology change can be seen by the ventral layer, where the nail matrix is degraded and less uniform. In the dirty specimen (right panel), darker areas are observed at the edge of the ventral layer. For orientation, corresponding regions in a) and b) are highlighted in pink.

c) RGB images for samples 8b3 and 8a2, showing Co (red), S (green) and Fe (blue). The dark green regions are areas of low S, coinciding with the areas of degraded keratin. The magenta-coloured regions have no S and originate from the overlap of Co (red) with Fe (blue); they correspond to the darker regions observed in a) and b), likely indicating deposition of external contamination onto the nail. Less contamination is present on the washed sample (left), if compared to the dirty specimen (right).

#8 traverse section (dirty)

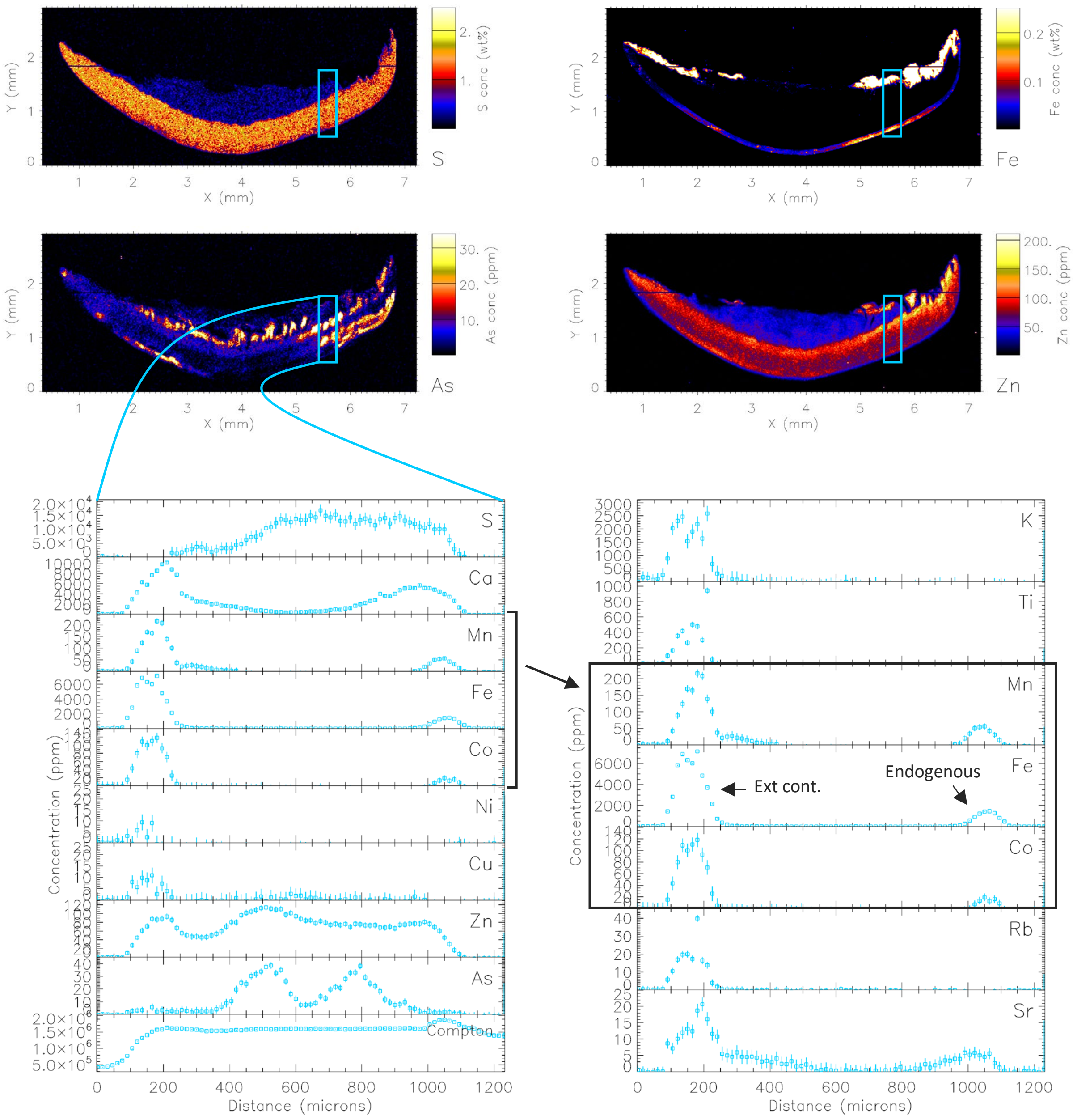

Figure S6. The cyan traverse section was drawn in an area with a visible distinction between endogenous and exogenous incorporation of Co, Mn, and Fe. The high levels of these metals observed in proximity of the ventral layer correlate with high concentrations of K, Ti, and Rb, whereas the concentration peaks found in the dorsal layer are not associated with any of the elements indicative of external contamination.

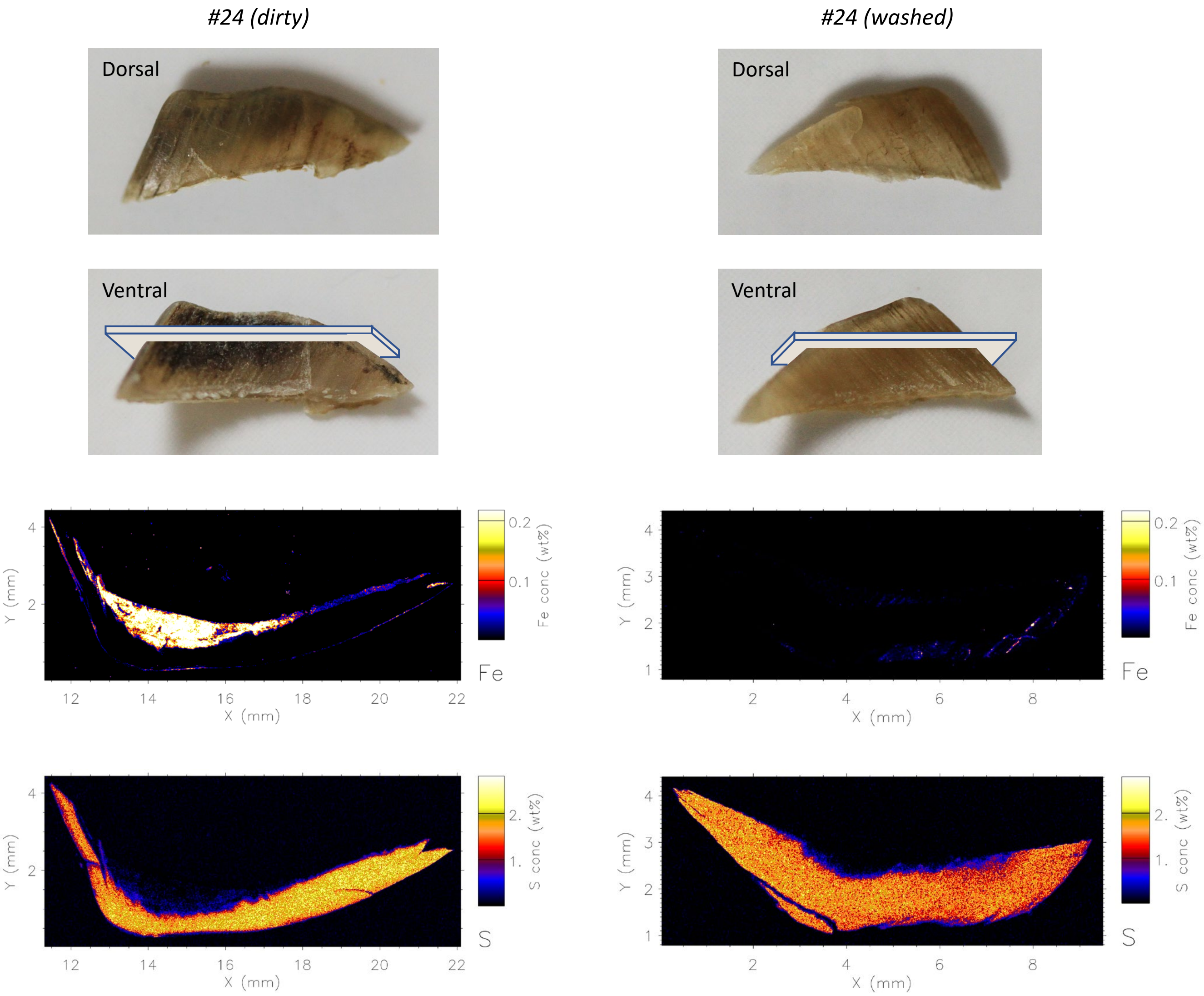

Figure S7. XFM maps of the measured trace elements in a thin section of the nails of participant #24 (dirty, left versus washed, right). In contrast to the sample in Figure S8, these XFM maps are indicative of the effectiveness of the cleaning protocol in removing loosely attached exogenous contamination. Prior to the washing steps, the two toenails were similar, with high levels of external material deposited onto the ventral side. The dark areas in the dirty sample (left), highly enriched in Fe, do not persist in the washed sample (right) after the cleaning steps.

#15 (washed)

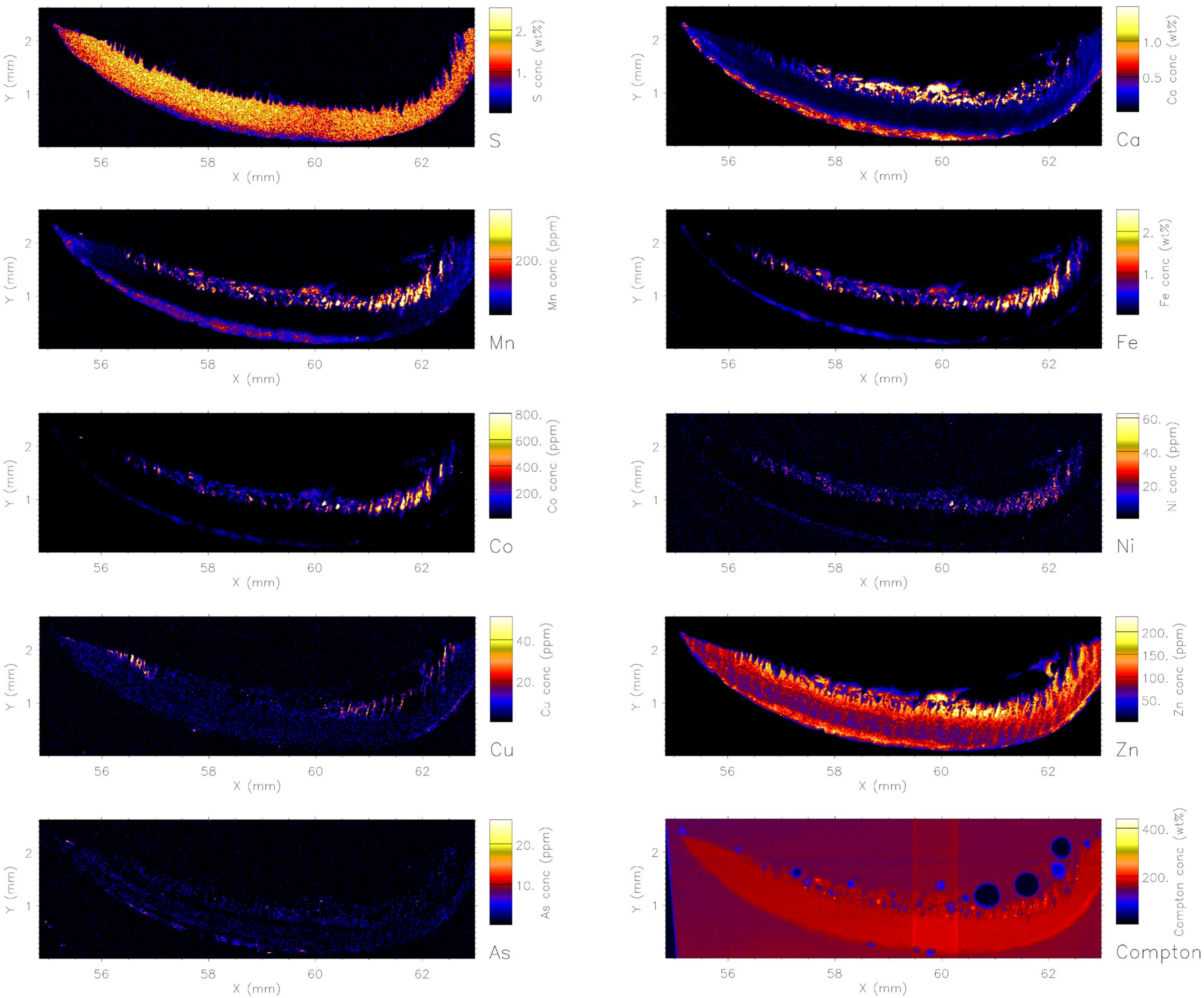

Figure S8. XFM maps of the measured trace elements in a thin section of the washed nail of participant #15. This sample has a very irregular and undulated ventral layer, in proximity of which there is a strong build-up of external contamination, despite having applied the washing protocol.

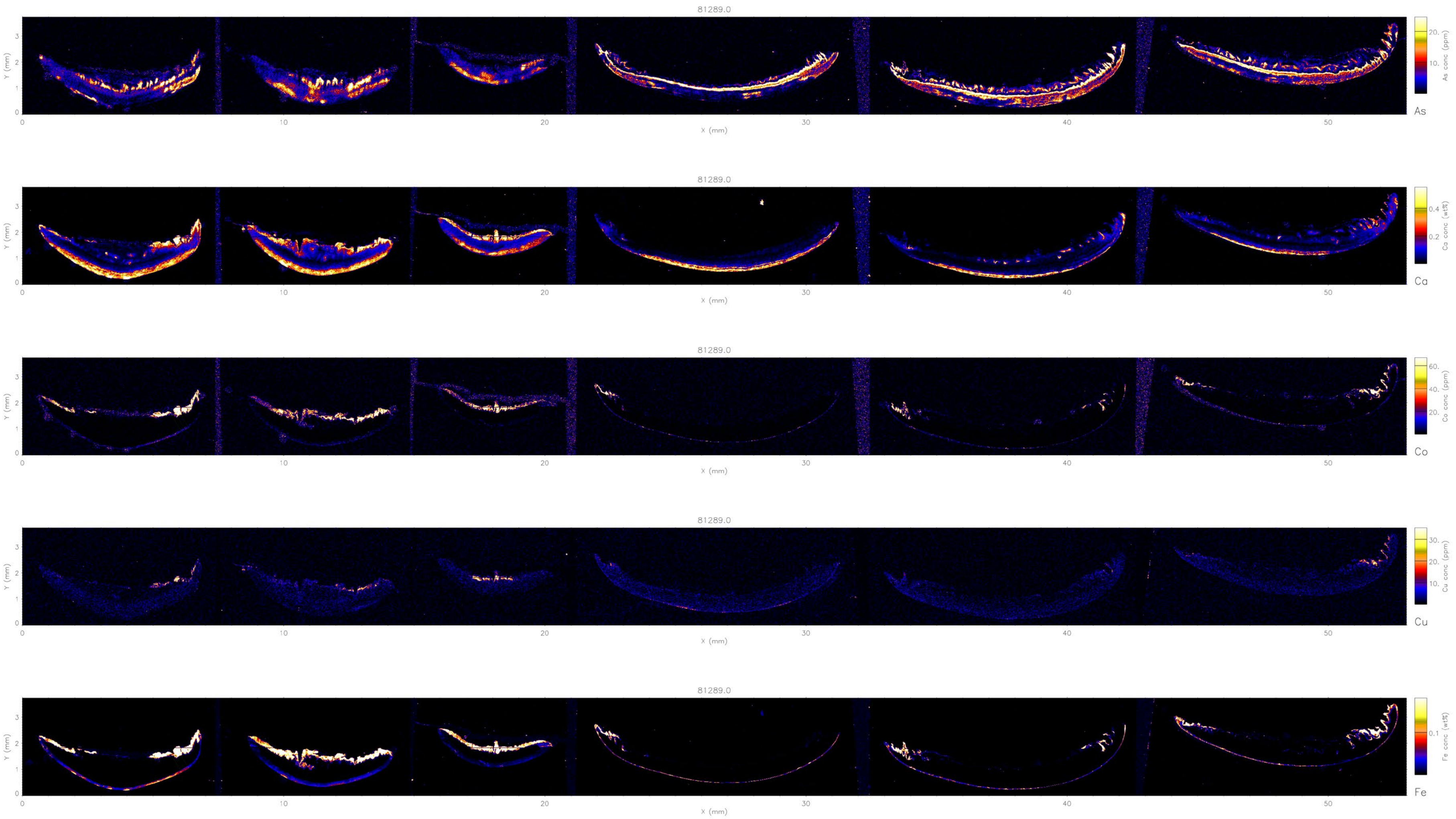

Figure S9. XFM maps for multiple transversal thin sections of participant #8 (dirty first three from the left, and washed last three from the left). Trace element order: As, Ca, Co, Cu, and Fe.

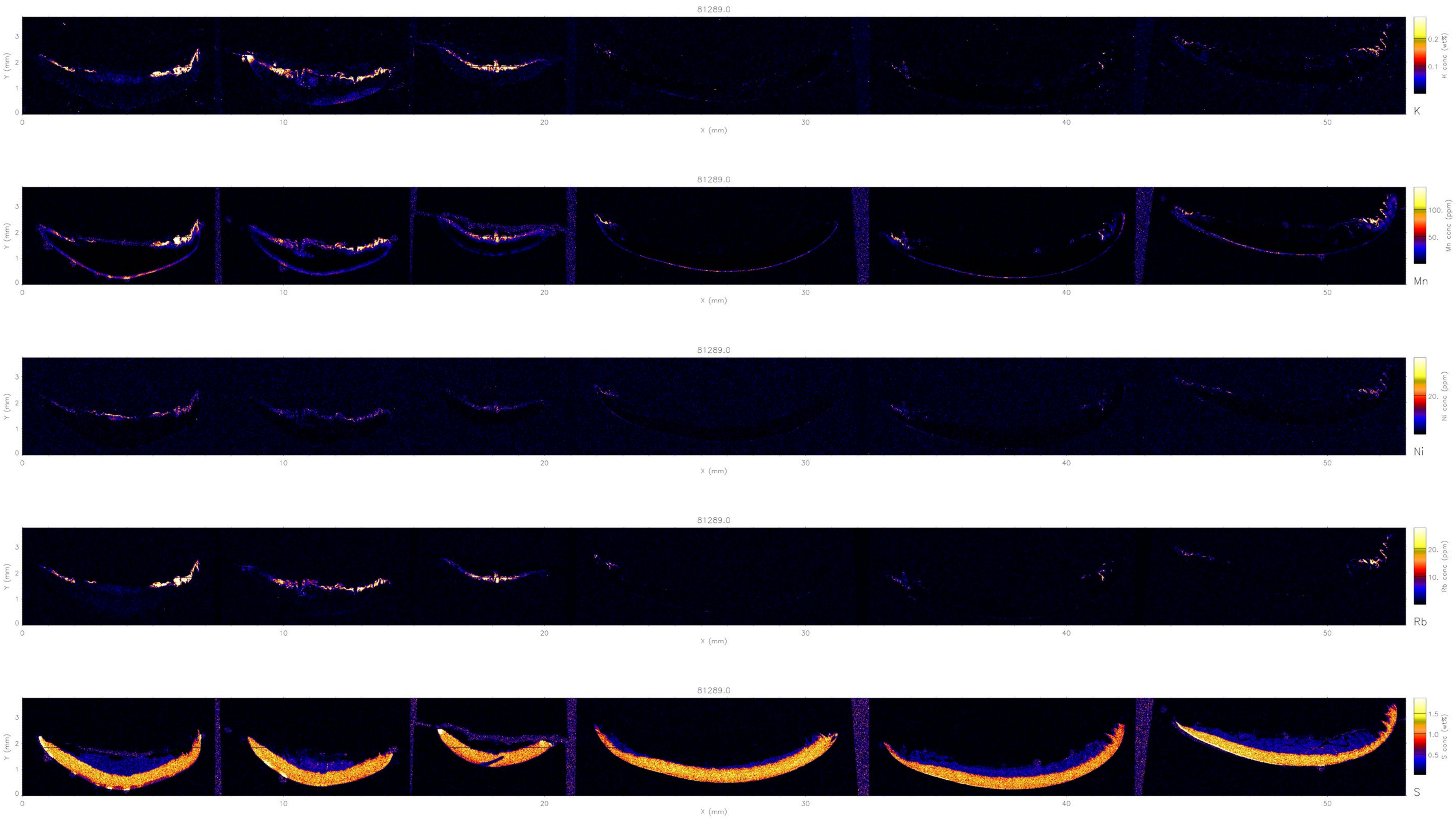

Figure S9 (continued). XFM maps for multiple transversal thin sections of participant #8 (dirty first three from the left, and washed last three from the left). Trace element order: K, Mn, Ni, Rb, and S.

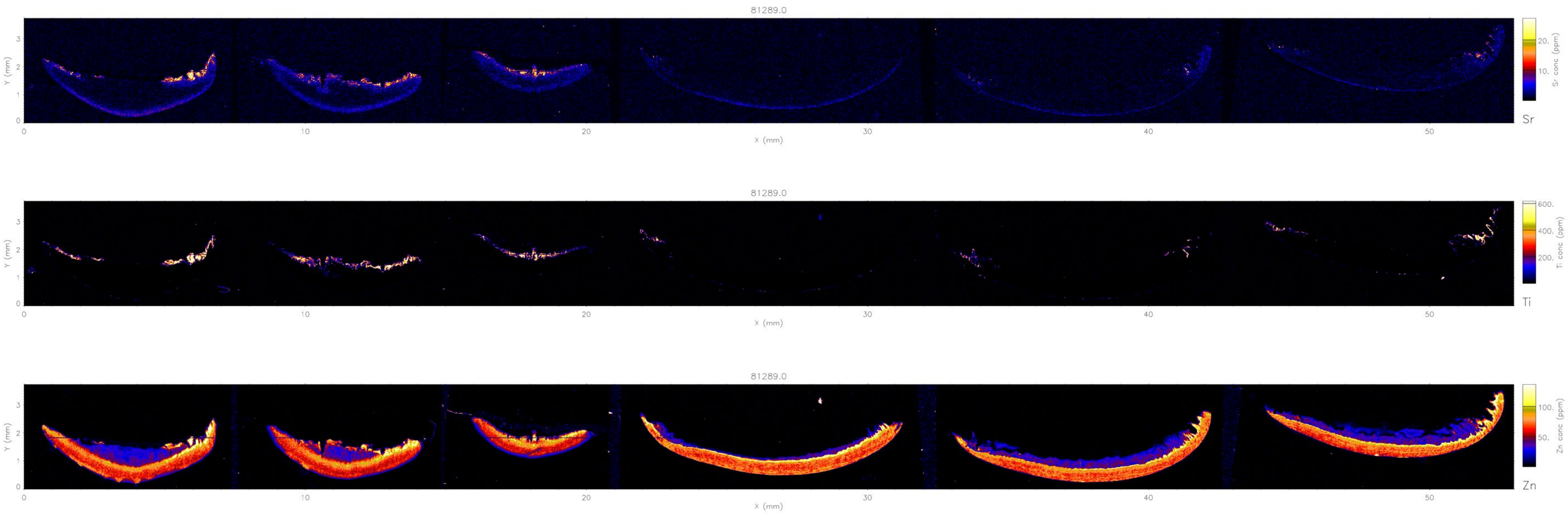

Figure S9 (continued). XFM maps for multiple transversal thin sections of participant #8 (dirty first three from the left, and washed last three from the left). Trace element order: Sr, Ti, and Zn.

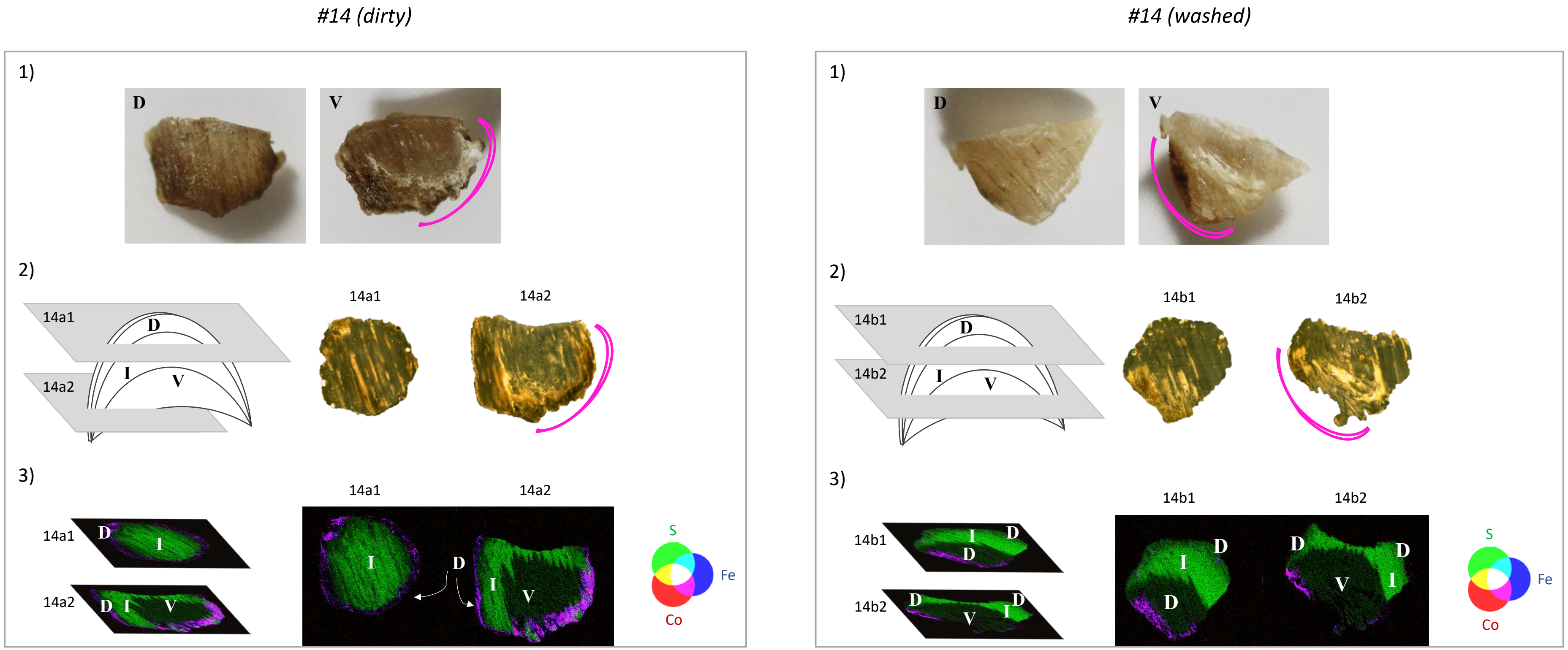

Figure S10. Not all thin sections were obtained by cutting the nails transversally. Some samples were also cut using different orientations, e.g., longitudinally (parallel to the nail bed). In these instances, similar patterns to those described earlier were observed. For example, lower S concentrations (dark green areas) are present in the dorsal and ventral layers of the nails of participant #14 (dirty, left panel versus washed, right panel). In the dirty sample (left panel), external contamination forms a rim onto the ventral layer, which is visible in the whole toenail pictures, in the microscope images, and in the RGB images where the overlap of Co and Fe generates a pink colour. Lower amounts of dirt are present on the washed sample (right panel), which however has a build-up of degraded keratin by the ventral layer.

1) Whole toenails from participant #14: dirty, left and washed, right, with their respective dorsal (D) and ventral (V) sides facing up.

2) The schematics of how the thin sections were obtained from the whole toenails, with the corresponding imaging and layer identification (D = dorsal, I = intermediate, V = ventral) shown in 3). On the right, optical microscope images of the thin sections. For orientation, corresponding regions in 1) and 2) are highlighted in pink.

3) RGB images of the thin sections, showing Co (red), S (green), and Fe (blue). The dark green regions are areas of low S. The pink-coloured regions originate from the overlap of Co (red) with Fe (blue).

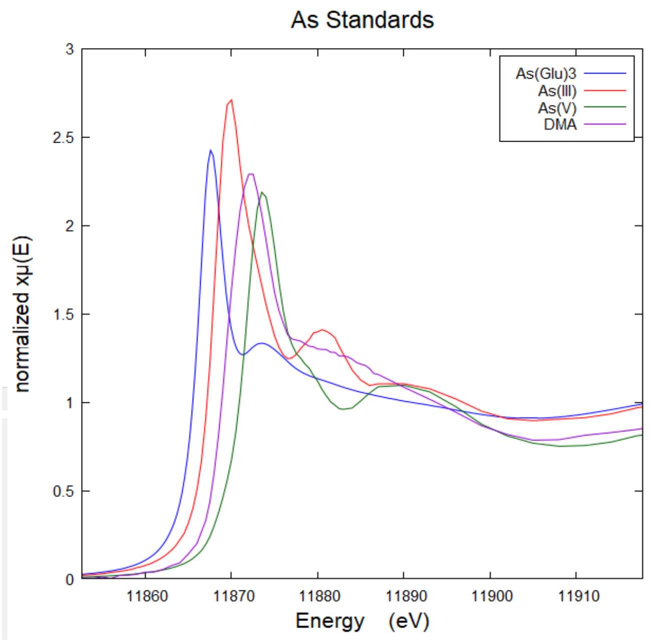

Figure S11. As K-edge XANES spectra of the four standards used for analysis: As-glutathione $As^{III}(GS)_3$ (11868.5 eV), arsenite As^{III} (11870.0 eV), DMA (11871.5 eV), and arsenate As^{V} (11873.5 eV). The spectra were background- and baseline-corrected using the Athena software (version 0.9.26).[1]

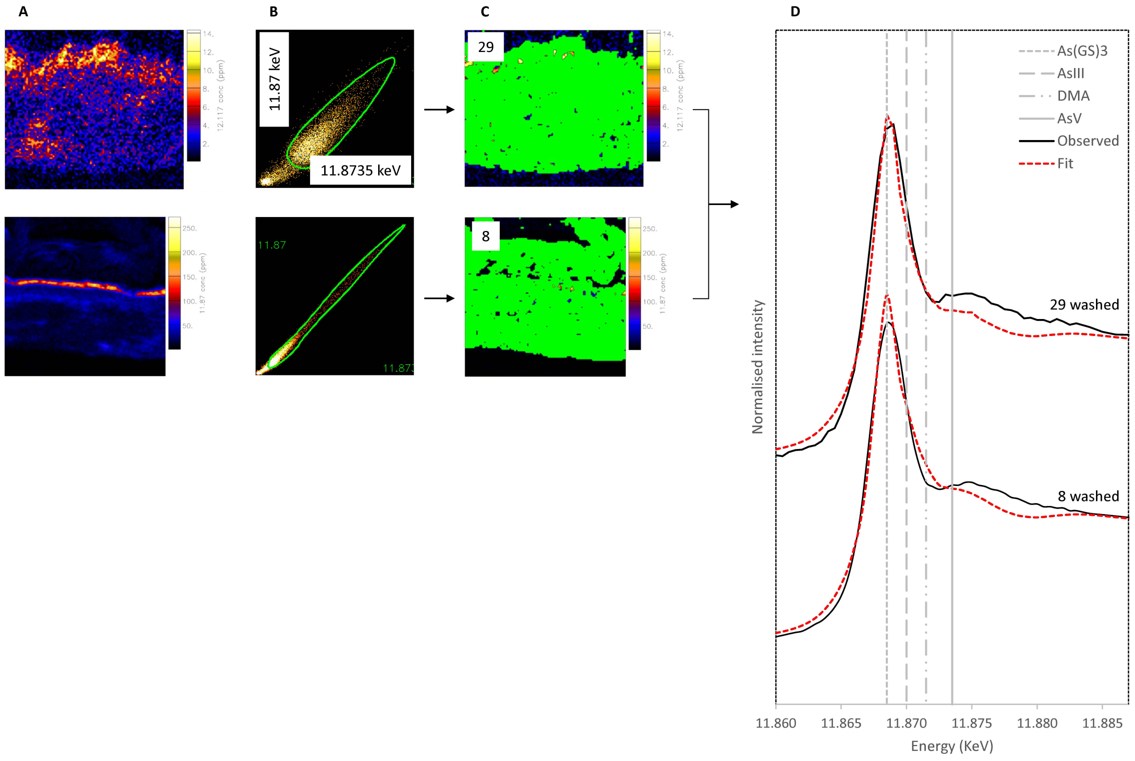

Figure S12. (A) Nail region of participants #29 and #8 selected for XANES (washed samples). (B) Energy association scatter plots corresponding to energies near the white line peak of the As^{III}–S bond in the nail specimen. (C) Localisation of the populations of energy correlations. (D) Extracted XANES for the populations; the vertical lines correspond to the white line peaks of $As^{III}(GS)_3$, As^{III}, DMA, and As^{V} standards.

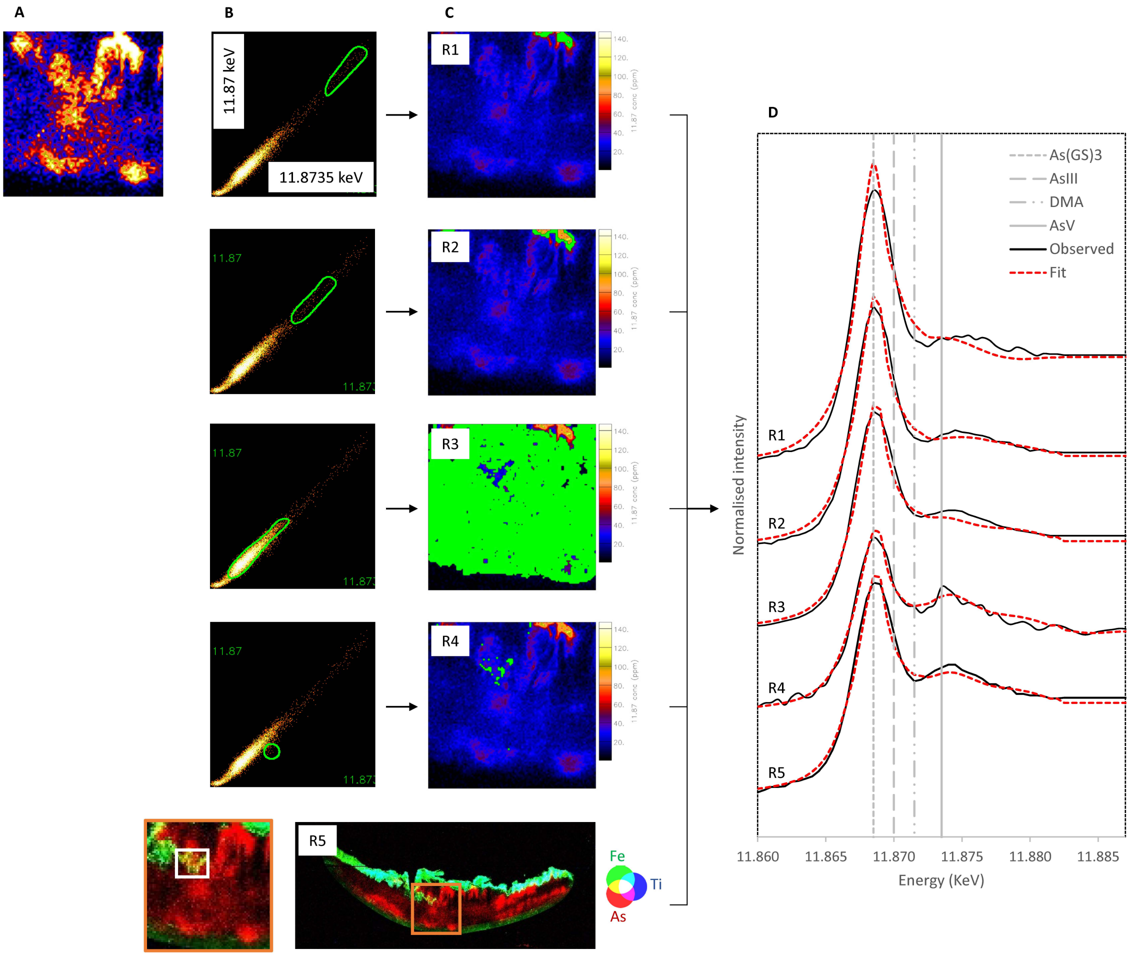

Figure S13. (A) Nail region of participant #8 selected for XANES (dirty sample). (B) Energy association scatter plots corresponding to energies near the white line peaks of the As^{III}–S bond in the nail specimen and of the As^{V} standard. (C) Localisation of different populations of energy correlations. (D) Extracted XANES for the five different populations; the vertical lines correspond to the white line peaks of $As^{III}(GS)_3$, As^{III}, DMA, and As^{V} standards.

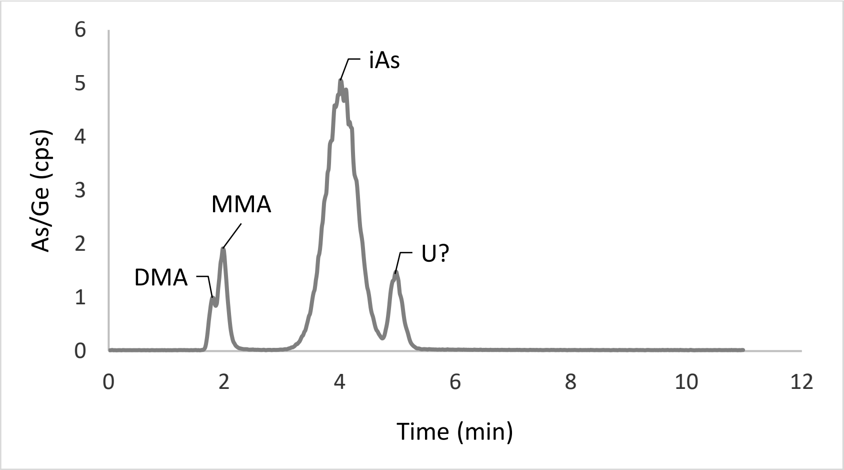

Figure S14. HPLC-ICPMS chromatogram showing the As content in participant #8. Eluting order: DMA (0.422 mg/kg), MMA (0.707 mg/kg), inorganic As, and unknown (8.910 mg/kg [iAs and unknown combined]). The retention time shift of iAs compared to its usual retention time (see below) is possibly due to heavy matrix effects. Instead, the presence of an additional peak (labelled U?) could be iAs as thioarsenate or arsenite eluting after the main iAs peak due to the heavy matrix in this particular sample.

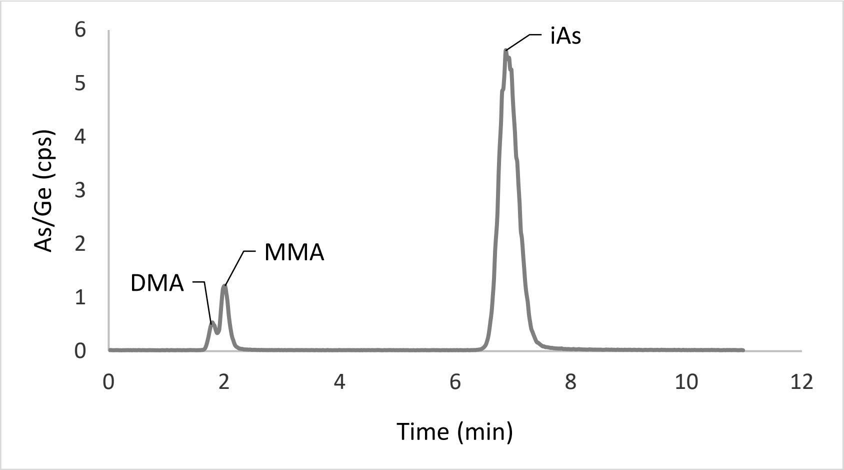

Figure S15. HPLC-ICPMS chromatogram showing the As content in participant #29. Eluting order: DMA (0.286 mg/kg), MMA (0.709 mg/kg), inorganic As (7.789 mg/kg).

References

1 Ravel, B., Newville, M. ATHENA, ARTEMIS, HEPHAESTUS: data analysis for X-ray absorption spectroscopy using IFEFFIT. *J. Synchrotron Radiat.* 2005, **12**, 537–541.
